# North American Wetlands and Mosquito Control

**DOI:** 10.3390/ijerph9124537

**Published:** 2012-12-10

**Authors:** Jorge R. Rey, William E. Walton, Roger J. Wolfe, Roxanne Connelly, Sheila M. O’Connell, Joe Berg, Gabrielle E. Sakolsky-Hoopes, Aimlee D. Laderman

**Affiliations:** 1 Florida Medical Entomology Laboratory and Department of Entomology and Nematology, University of Florida-IFAS, Vero Beach, FL 342962, USA; E-Mails: crr@ufl.edu (R.C.); sheilao@ufl.edu (S.M.O.C.); 2 Department of Entomology, University of California, Riverside, CA 92521, USA; E-Mail: walton@ucr.edu; 3 Connecticut Department of Energy and Environmental Protection, Franklin, CT 06254, USA; E-Mail: roger.wolfe@ct.gov; 4 Biohabitats, Inc., 2081 Clipper Park Road, Baltimore, MD 21211, USA; E-Mail: jberg@biohabitats.com; 5 Cape Cod Mosquito Control Project, Yarmouth Port, MA 02675, USA; E-Mail: gsakolsky@aol.com; 6 Marine Biological Laboratory, Woods Hole, MA 02543, USA; E-Mail: aladerman@gmail.com

**Keywords:** arbovirus, marsh, mangrove, mosquito control, surveillance, wetland

## Abstract

Wetlands are valuable habitats that provide important social, economic, and ecological services such as flood control, water quality improvement, carbon sequestration, pollutant removal, and primary/secondary production export to terrestrial and aquatic food chains. There is disagreement about the need for mosquito control in wetlands and about the techniques utilized for mosquito abatement and their impacts upon wetlands ecosystems. Mosquito control in wetlands is a complex issue influenced by numerous factors, including many hard to quantify elements such as human perceptions, cultural predispositions, and political climate. In spite of considerable progress during the last decades, habitat protection and environmentally sound habitat management still remain inextricably tied to politics and economics. Furthermore, the connections are often complex, and occur at several levels, ranging from local businesses and politicians, to national governments and multinational institutions. Education is the key to lasting wetlands conservation. Integrated mosquito abatement strategies incorporate many approaches and practicable options, as described herein, and need to be well-defined, effective, and ecologically and economically sound for the wetland type and for the mosquito species of concern. The approach will certainly differ in response to disease outbreaks caused by mosquito-vectored pathogens versus quality of life issues caused by nuisance-biting mosquitoes. In this contribution, we provide an overview of the ecological setting and context for mosquito control in wetlands, present pertinent information on wetlands mosquitoes, review the mosquito abatement options available for current wetlands managers and mosquito control professionals, and outline some necessary considerations when devising mosquito control strategies. Although the emphasis is on North American wetlands, most of the material is applicable to wetlands everywhere.

## Acronyms

BMP: Best Management Practices
Bti: *Bacillus thuringensis* var. *israelensis*
EEE: Eastern Equine Encephalitis
FIFRA: Federal Insecticide, Fungicide, and Rodenticide Act
FWS: Free Water Surface
USFWS: U.S. Fish and Wildlife Service
IGR: Insect Growth Regulator
IMM: Integrated Mosquito Management
IPN: Integrated Pest Management
Ls: *Lysinibacillus sphaericus*
MMF: Monomolecular Film
NOI: Notice of Intent
NPDES: National Pollutant Discharge Elimination System
OMWM: Open Marsh Water Management
PNW: Pacific Northwest
RIM: Rotational Impoundment Management
RRV: Ross River Virus
SLE: St. Louis Encephalitis
SSF: Sub-surface Flow
ULV: Ultra-low Volume
VEE: Venezuelan Encephalitis
WEE: Western Equine Encephalitis
WN: West Nile
WNV: West Nile Virus

## 1. Introduction

Wetlands are valuable habitats that provide important social, economic, and ecological services such as flood control, water quality improvement, carbon sequestration, pollutant removal, and primary/secondary production export to terrestrial and aquatic food chains. They represent important habitats for a large number of animal and plant species, some of which are threatened or endangered. Wetlands protect adjacent habitats from erosive forces, and have important flood control and water storage functions. Wetlands also have high aesthetic and recreational value which makes neighboring areas highly desirable for human habitation. However, wetlands are also natural producers of mosquitoes and this sometimes creates conflicts with human neighbors.

Most frequently, wetlands mosquito production is a “nuisance” issue, affecting the quality of life of nearby residents by instigating generally undesired behaviors. Examples include postponement or cancellation of pleasurable activities such as hikes, picnics, and other forms of outdoor recreation; necessity to apply repellent while outdoors; or simply having to endure uncomfortable and irritating mosquito bites during the course of normal or extracurricular activities. Large biting mosquito populations can sometimes also have social, cultural, and economic impacts by limiting community activities. Examples include cancellation of, or reduced attendance to revenue-generating activities such as concerts and sporting events; drops in tourism; and reduction in outdoor activities that drive local economies and support local merchants (e.g., hunting, fishing, hiking, *etc.*) [1,2,3].

Mosquitoes can have grave health impacts on the population at large when mosquito-transmitted pathogens such as West Nile virus, eastern equine encephalitis virus, and *Plasmodium* spp. (the causative agent of malaria) are being amplified and transmitted locally. Mosquitoes can also routinely have serious health impacts on individuals with allergies to mosquito bites [4]. Severe reactions to mosquito bites can be local or systemic and can cause tissue necrosis, urticaria, inflammation of the mucous membranes, fever, lowering of blood pressure, loss of consciousness and other symptoms [5,6]. Mosquitoes can also have significant health impacts on wildlife, livestock, and pets including wild birds, cattle, dogs, and horses [7,8].

There is disagreement about the need for mosquito control in wetlands and about the techniques utilized for mosquito abatement and their impacts upon wetlands ecosystems. For example, some authors [9] consider permanent and semi-permanent source reduction techniques to be suitable only for wetlands already heavily impacted by human activities or for intensely managed wetlands, whereas others consider some of these techniques to be a form of marsh restoration [10,11]. Furthermore, public misconceptions abound about the role of wetlands in local mosquito production and disease transmission, and on the ecological impacts of particular mosquito control activities even though published research does not support such misconceptions.

There is often a lack of information on wetlands ecology and management in the mosquito control literature, but the opposite is also true; information on mosquitoes, mosquito-borne pathogens, and mosquito control technology is also lacking in the wetlands, conservation, restoration, and wetlands/water management literature [12]. In fact, even the wetlands literature is often fragmented by wetlands types (e.g., freshwater swamps *vs.* coastal wetlands), with few insights forthcoming from patterns and processes common to many wetland types [13]. We consider this situation to be a critical shortcoming in our ability to deal with wetlands mosquito production in an effective and ecologically sound manner.

In this contribution, we provide an overview of the ecological setting and context for mosquito control in wetlands, present pertinent information on wetlands mosquitoes, review the mosquito abatement options available for current wetlands managers and mosquito control professionals, and outline some necessary considerations when devising mosquito control strategies. We also provide relevant literature for wetlands practitioners and mosquito control/public health personnel and discuss pressing research needs. We encourage research on ecologically-sound mosquito abatement techniques and on strategies and policies that further the above goal.

## 2. Types of Wetlands

**Figure 1:**
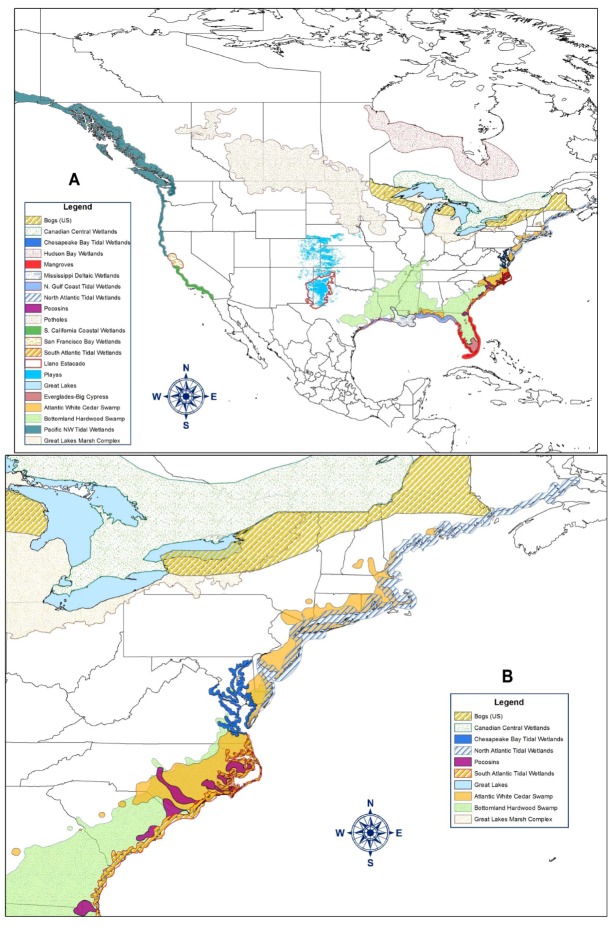
Map showing the distribution of some of the wetlands types discussed in the paper. Outlines show general location and not actual coverage. (**A)** North America; (**B)** United States East Coast north of Florida (approximately 30.5°N lat.).

Below, we briefly describe the major types of wetlands in North America to establish the context and scope of mosquito control-wetlands issues (Table 1). It is important to note that many variations for a particular wetland “type” occur and that in reality, no two wetlands are exactly alike. It is not possible to cover every single wetland type [13], but we expect that most, including many highly localized types can be included as subtypes of the wetlands listed here. Figure 1 shows the general location of some of these wetlands. There are several excellent references that offer more detailed discussion on the different wetland types. Included among these are: Mitsch and Gosselink [13], Batzer and Sharitz [14], Perillo *et al*. [15], Batzer and Baldwin [16]. Sub-tidal areas such as seagrass beds and high energy coastlines are not included because they normally do not provide suitable larval habitats for mosquitoes.

**Table 1:** Summary of wetland types discussed in the text.

Type	USFWS^ 1^ classification	Major hydrologic influence	Flooding frequency
Mangrove	Estuarine forested/shrub	ocean tide	daily-seasonal
Tidal salt marsh	Estuarine intertidal emergent	ocean tide	daily-seasonal
Pacific Northwest tidal wetland	Estuarine intertidal emergent	ocean tide	daily-seasonal
Tidal brackish marsh	Estuarine intertidal emergent	tide/surface	daily-seasonal
Tidal freshwater wetland	Palustrine emergent	surface	daily-seasonal
Bottomland swamp	Palustrine emergent/ forested	river, precipitation, ground	semi-permanent
Atlantic white cedar wetland forest	Palustrine emergent/ forested	precipitation, ground	seasonal^ 2^
Riverine riparian floodplain wetland	Palustrine emergent/ forested	river, precipitation,	variable
Wet meadow	Palustrine emergent	ground	seasonal
Wet prairie	Palustrine emergent	ground	permanent, semi-permanent^ 2^
Playa	Palustrine emergent	surface	seasonal
Bog	Palustrine shrub	precipitation/runoff	variable/seasonal^ 2^
Pocosin	Palustrine shrub	ground	semi-permanent^ 2^
Fen	Palustrine shrub	ground	semi-permanent^ 2^
Carolina Bay	Palustrine shrub	precipitation/ground	permanent-seasonal
Pothole	Pond natural	precipitation	variable
Vernal pool	Pond natural	precipitation	seasonal
Mississippi deltaic plain wetlands	Mixed	various	variable
Everglades	Mixed	various	variable
Constructed wetlands	Mixed	-	permanent

^1 ^U.S. Fish and Wildlife Service; ^2 ^Includes saturated surface with or without standing water.

### 2.1. Coastal Wetlands

Coastal wetlands include a variety of ecosystems influenced in some way by ocean tides. Here we consider mangrove forests, tidal salt marshes, Pacific Northwest tidal wetlands, tidal brackish marshes, and tidal freshwater wetlands. Wolanski *et al.* [17] estimate worldwide coverage of mangroves, and freshwater wetlands to be 230,000 and 300,000 km^2^, respectively. North American salt marsh cover is close to 300,000 km^2^ [13]. These ecosystems are particularly vulnerable to human impacts because of the desirability of the habitat for human habitation and also to effects of sea level changes due to their geographic location.

#### 2.1.1. Mangroves

The term mangrove refers to assemblages of tropical trees and shrubs that grow in the intertidal zone. It is a non-taxonomic term used to describe a diverse group of plants that share certain ecological characteristics including adaptation to wet saline habitats. Terms such as mangrove community, mangrove forest, mangrove swamp, mangrove wetland, and mangal are used interchangeably to describe the entire mangrove community. They occur worldwide along tropical and subtropical coasts, in areas with unconsolidated sediments and low to moderate wave action [18]. In the United States (U.S.) mangroves are found principally in Florida south of 30°N latitude, predominantly in the Florida Keys, southwest Florida, and southeast/east-central Florida (Figure 1). As with other coastal vegetation, mangal hydrology is primarily influenced by tides [18]. Freshwater inputs from precipitation and upland runoff can be important and can play important roles in modifying salinity regimes, nutrient cycling, and flushing [19].

Worldwide there are approximately 40 to 50 species of mangroves distributed among 15–16 families of plants. Three mangrove species occur in Florida: the red mangrove (*Rhizophora mangle*), the black mangrove (*Avicennia germinans*), and the white mangrove (*Laguncularia racemosa*). A variety of herbaceous halophytes often occupy the mangrove understory. Examples include *Spartina alterniflora*, *Batis maritima*, *Salicornia virginica*, and *Distichlis spicata.*

Mangrove forests serve as important permanent habitat, and as foraging, refuge, and breeding/nursery habitat for a large number of terrestrial and aquatic organisms including several threatened or endangered species. Many of these species are highly valued by sports and commercial fisheries, inshore and offshore. Marine invertebrates such as crustaceans, mollusks, and worms, terrestrial invertebrates (insects and arachnids), reptiles (e.g., the American crocodile, *Crocodylus acutus*), and a multitude of birds are common in mangrove forests. Many important fish species such as snapper (*Lutjanus* spp.) and tarpon (*Megalops atlanticus*) depend on these areas during part of their life cycle. Mammals known to frequent mangrove areas include the West Indian Manatee (*Trichechus manatus*) and the endangered Florida Key Deer (*Odocoileus virgininaus clavium*).

Mangroves are well known for their high primary production and are extremely important for nutrient dynamics of adjoining estuaries. Although the role of mangroves as contributing to “land building” is doubtful, mangroves do serve as barriers to shoreline erosion, and help to stabilize sediments thus reducing the effects of storm surges and heavy surf. Mangroves also help improve water quality by extracting excess nutrients and by facilitating the detoxification and storage of pollutants in the sediments. Mangroves are prime areas for outdoor recreational activities such as fishing, boating, sightseeing, wildlife observation, and many others.

Human impacts have resulted in major losses of mangrove habitat [19] Major impacts worldwide include clearing for residential, industrial, and commercial development; agriculture and charcoal production; salt extraction; and others. Pollution related to human development (residential; commercial, including tourism; and industrial), and nearby agriculture has also resulted in significant loss and degradation of mangrove habitats.

Mangrove wetlands can produce significant numbers of mosquitoes and because of topography and tidal phenomena a large proportion of the mangrove acreage in Florida is suitable for mosquito production [20,21]. For example, Carlson and Vigliano [22] report that 12 mosquito broods were produced during a mosquito season from a single quadrat (of unknown size) in an impounded mangrove wetland in Florida, with brood sizes of up to 349 immature mosquitoes per 350 mL sample. The primary mosquito species is the black salt marsh mosquito *Aedes taeniorhynchus*. This species is an aggressive biter and a strong flier, with potential impacts 30 km from the mangrove forests [23] and as far as 90 km under ideal conditions [24]. The species bites during both day and night and is an important vector of canine heartworm (*Dirofilaria immitis*) and Venezuelan encephalitis [25,26]. *Aedes taeniorhynchus* can transmit eastern equine and St. Louis encephalitis [27,28], and West Nile virus [28] but it has never been implicated as a major vector of these diseases in Nature. *Aedes sollicitans*, *Culex nigripalpus,* and *Culex salinarius* are infrequently collected in the less saline (brackish) sections of mangals (e.g., [22]).

#### 2.1.2. Tidal Salt Marshes

Tidal salt marshes are coastal wetlands that are regularly influenced by ocean tides. In very broad terms they can be divided into low salt marsh, which is influenced by daily tides, and high salt marsh, that is only flooded by spring tides and/or seasonal high tides. The high marsh is the most important habitat for mosquito production as the frequent inundation of low marshes usually prevents significant mosquito production from these areas [29]. Although these habitats can occur directly in front of open ocean when the wave energy is low (for example along the Gulf of Mexico coast), they more often occur behind protective barrier islands [13]. Many intertidal marshes have very high primary production [10] that is exported directly or as secondary production [30] to adjoining estuaries and coastal areas and supports marine and estuarine food webs. Marshes provide a variety of other services including flood control, water storage, erosion prevention, water quality enhancement and recreation.

In North America they are most abundant on the Atlantic Coast from Maine to Florida and along the Gulf Coast (Figure 1). Limited bands of salt marshes occur in Southern California and along the Pacific Northwest Coast (see below), expanding again along the coast of Alaska. In Florida, coastal salt marshes are gradually replaced by mangroves below 30°N latitude, but narrow spits of salt marsh can be found throughout the State. Hydroperiod is greatly influenced by the local tidal regime, with extensive tidal penetration with high amplitude lunar tides such as occur in regions of the Atlantic Coast of North America, to irregular low amplitude flooding by wind generated tides in the Gulf Coast and protected estuaries such as the Indian River Lagoon in Florida. Although ocean tides are the predominant hydrological influence, the balance between tidal and freshwater inflows is critical in determining marsh characteristics and function.

Above 30°N latitude on the Atlantic Coast, these marshes are usually dominated by halophytic grasses, rushes and succulents including several *Spartina* species particularly smooth cordgrass, (*Spartina alterniflora*) and saltmeadow cordgrass (*S. patens*), saltwort (*Batis maritima*), glasswort, (*Salicornia bigelovii, Sarcocornia perennis*), salt grass*,* (*Distichlis spicata*), saltmeadow rush (*Juncus gerardi*) and black needle rush (*J. roemerianus*) and many others. Several shrubby halophytes can also be found at the higher elevations of these marshes such as the upland edges and on top of natural or artificial berms and levees [31].These include groundsel, *Baccharis halimifolia*; marsh elder, *Iva frutescens*; and wax myrtle, *Myrica cerifera*.

Although the Pacific coast of North America is noted for its nearby mountains, steep bluffs, and rocky shores, many small, semi-isolated coastal salt marshes occur along river valleys, particularly south of Point Conception , approx. 34°26.7'N, 120°28.3'W; [32] (Figure 1). In these coastal wetlands, *Spartina foliosa* predominates in the low marsh and various succulents (e.g., *Salicornia * spp.) and other herbaceous species such as salt grass (*Distichlis spicata*) in the high marsh [32]. Other common S. California marsh species include saltwort, glasswort, marsh jaumea (*Jaumea carnosa*), California seablite (*Suaeda californica*), and many others. Pacific Northwest coastal wetlands are described below. 

As with mangrove forests, tidal salt marshes are essential foraging and nursery habitats for a great number of marine and estuarine species, including some of high commercial and/or recreational value such as snook (*Centropomus undecimalis*), tarpon (*Elops saurus*), and mullet (*Mugil* spp.). Many salt marsh areas along all North American coasts are extremely important habitat for waterfowl, shore and migratory birds [33] including many high priority species such as the salt marsh sharp-tailed sparrow (*Ammodramus caudacutus*), clapper rail (Rallus longirostris), and the roseate spoonbill (*Platalea ajaja*). In addition to a multitude of marine invertebrates, tidal wetlands are home to numerous terrestrial vertebrates including turtles, frog, snakes, alligators; and mammals such as deer (*Odocoileus virginianus )*, raccoon (*Procyon lotor*), otters (*Enhydra lutris*), dolphins (*Tursiops* spp.), manatees (*Trichechus manatus*) and others [34,35].

More than half of the historic salt marsh habitat of the United States has been lost, in great part due to human activities [36]. Major environmental impacts to coastal salt marshes are related to residential, agricultural, and industrial development and include direct habitat loss, habitat fragmentation, and contamination. Eutrophication-related problems such as noxious algal blooms and hypoxia are becoming more prevalent, particularly in the southern regions [34], and modification of freshwater flow and sediments by ditching, diking, and channelization of rivers have severely impacted coastal wetlands, particularly in the Gulf region [37]. Many salt marshes can also produce vast numbers of mosquitoes. For example, James-Pirri *et al.* [38] report larval densities of up to 130 per 350 mL dip in New Jersey (USA) marshes already grid ditched for mosquito control, and in California coastal wetlands, more than 10,000 adult California salt marsh mosquitoes, *Aedes squamiger*, have been collected in a single night in one Fay trap [39]. The major salt marsh mosquito species in the Atlantic and Gulf Coasts include the tan salt marsh mosquito *Aedes sollicitans*, the black salt marsh mosquito *Aedes taeniorhynchus*, and the more northern brown salt marsh mosquito *Aedes cantator*. Along the Pacific Coast, the California salt marsh mosquito and the summer salt marsh mosquito *Aedes dorsalis* predominate. The Florida SLE (St. Louis Encephalitis) vector *Culex nigripalpus* and the un-banded salt marsh mosquito *Culex salinarius* may occur infrequently along the brackish upper marsh borders of the Atlantic and Gulf coasts whereas the winter marsh mosquito *Culiseta inornata* and *Culex tarsalis* (another encephalitis virus vector) can occur in the same general locations along the Atlantic, Gulf, and Pacific Coasts.

#### 2.1.3. Pacific Northwest Tidal Wetlands

Pacific Northwest (PNW) tidal wetlands (including northern California) occur in a geologically and topographically diverse area. The usually steep topography of this area presents a limited range for these wetlands [40], and results in a large number of small isolated or semi-isolated estuaries and coastal wetlands (Figure 1). Due to the hydrological forces that create them, individual wetlands in the region have unique characteristics that vary by estuary type and landscape, although there are still some basic common characteristics [41].

In addition to the direct tidal influence, the marshes in the major watersheds in the Pacific NW are heavily influenced by the discharge plumes of associated river systems For example, Puget Sound with discharges from the Nooksack, Dungeness, and Elwha Rivers, Willapa Bay from the Bone, Niawiakum, Palix , Naselle, Bear, and Willapa rivers; and most of the northern region from the plume of the Columbia River. Additionally, precipitation in the area can vary significantly with annual amounts generally increasing from south to north, but with considerable local variation associated with local characteristics such as mountain rain shadows [42].

In general, low marshes are dominated by halophytic succulents such as *Salicornia virginica*, *Jaumea carnosa*, and *Triglochin maritima* as well as several grasses and sedges such as *Distichlis spicata* and Lyngbye’s sedge *Carex lyngbyei*. Tufted hairgrass (*Deschampsia caespitosa*) commonly dominates in high marshes, accompanied by mixes of many other species including Pacific silverweed (*Argentina egedii*) and Baltic rush (*Juncus arcticus* var. *balticus*). In some areas fresh water-influenced systems may have extensive communities of softstem and/or hardstem bulrush (*Schoenoplectus tabernaemontani* and *S. acutus*) [41].

The once common Pacific NW tidal forested wetlands are now rare due to anthropogenic impacts. Major tree/shrub species in the remaining areas include Sitka spruce (*Picea sitchensis*), black twinberry (*Lonicera involucrata*), and Pacific crabapple (*Malus fusca)*, with alder (*Alnus rubra*), willows (*Salix* spp.), Douglas spiraea (*Spiraea douglasii*), and colonial dogwood (*Cornus sericea*) often found in freshwater influenced areas. 

PNW tidal wetlands support diverse coastal wetland fish and invertebrate communities. Of special importance in this area are anadromous salmonids such as steelhead (*Onchorhynchus mykyss*) and various salmon species including Chinook (*O. tshawsytscha*), chum (*O. keta*), coho (*O. kisutch*), and pink (*O. gorbusha*) which use coastal wetlands during parts of their life cycle. These wetlands also provide habitat for numerous waterfowl, shorebirds, rail, including the endangered California clapper rail (*Rallus longirostris obsoletus*), and numerous raptors such as northern harriers (*Circus cyaneus*), osprey (*Pandion haliaetus*) and bald eagles (*Haliaeetus leucocephalus*). PNW tidal marshes occur along the Pacific Flyway and include many important Flyway sites such as Morro Bay, San Francisco Bay, and Padilla Bay [42]. Mammals regularly utilizing these wetlands range from mice and shrew to North American elk (*Corvus elaphus*).

Coastal wetlands of the Pacific NW have been extensively modified. For example, within California, close to 90% of historical coastal wetlands have been lost [43], Puget Sound has lost 70 to 80% of its estuarine marshes, and close to 70% of the Columbia River Estuary tidal wetlands no longer exist [42]. Approximately 94% and 77% of forested wetland losses had been reported by [44].

Land conversion for development, industry, and agriculture has eliminated many hectares of productive wetlands and has resulted in subsidence, contamination, and degradation of many others. Modification to reduce flooding and lower salinities (diking, ditching, culverts and tide gates) have changed the character of much of the remaining acreage. Common mosquitoes in this habitat include *Aedes squamiger*, and *Aedes dorsalis*.

#### 2.1.4. Tidal Brackish Marshes

Tidal brackish marshes form a transition between salt marshes and tidal freshwater marshes. They are found along all coasts and occur upstream of the salt marsh proper or at the mouth of coastal rivers with high freshwater discharge. There is often a gradation of marsh types with salinity, from salt marshes at the coast to freshwater tidal wetlands upland. Some classification systems divide brackish tidal marshes into oligohaline (less than 5 ppt salinity) and mesohaline (less than 18 ppt salinity). Tidal brackish marshes are only irregularly flooded by tidal waters and receive considerable fresh water inputs from rivers and streams, and overland runoff.

Although they share many plant species with the tidal salt marshes, brackish marshes tend to have higher plant diversity and lower species dominance, although localized monospecific vegetation stands are common. Depending upon location, characteristic plants may include saltmeadow cordgrass (*Spartina patens*), salt grass (*Distichlis spicata*), black needlerush (*Juncus roemerianus*) or saltmeadow rush (*Juncus gerardi*), chairmaker’s bullrush (*Schoenoplectus americanus*), salt marsh bulrush (*S. robustus*), dwarf spikesedge *(Eleocharis parvula*), seashore paspalum (*Paspalum vaginatum*), coastal water hyssop (*Bacopa monnieri*) saltbushes (*Atriplex* spp.), threesquare bulrush (*Schoenoplectus pungens*), big cordgrasss (*Spartina cynosuroides*) and cattails (*Typha* spp.). In West Coast brackish marshes *Typha* spp. *Schoenoplectus acutus* (= *Scirpus acutus*), *Atriplex prostrata* (= *Atriplex triangularis*) and *Phragmites* spp. often dominate.

Tidal brackish marshes are very important habitats for immature forms of many marine and estuarine organisms. On the mesohaline end, brackish marshes provide excellent habitat to typical estuarine animals such as blue crabs (*Callinectes sapidus*), redfish (*Sciaenops ocellatus*), spotted seatrout (*Cynoscion nebulosus*), fiddler crabs (*Uca* spp.), and many others. Near the oligohaline end, one finds species more typical of the freshwater marshes (see below).

Because of the irregular flooding, brackish marshes can also be significant mosquito producers. Depending on the location on the marsh, mosquitoes normally found in salt marshes such as *Aedes taeniorhynchus*, *A. sollicitans*, and *Culex salinarius*, and others more typical of fresh water areas including several *Culex*, *Anopheles*, and *Psorophora* species can be produced.

#### 2.1.5. Tidal Freshwater Wetlands

Tidal freshwater wetlands occur at the head of tide in coastal areas. Salinity is less than 0.5 ppt, the flora and fauna are dominated by freshwater species, and they experience regular tidal fluctuations.

Historically, the greatest expanses of tidal freshwater wetlands in North America occurred in the Atlantic coast between Georgia and New England [45] except where tidal amplitude is small such as estuaries protected by extensive barrier islands such as the Outer Banks of North Carolina. Steep, rocky coastlines in northern New England and Canada do not favor the development of extensive marshes, so in this area they tend to be small and isolated except for some large marshes along the St. Lawrence River watershed. Similar geomorphological conditions result in few tidal freshwater marshes along the Pacific coast of North America. Exceptions include extensive marshes in Alaska, in the Columbia River watershed, and along major river systems in California. Tidal freshwater wetlands also occur along the Gulf coast, but the low amplitude-mostly wind driven tides in this area result in much lower coverage and slightly different communities than in the Atlantic coast marshes [46].

Because tides can propagate upstream much farther than the salt water, freshwater tidal marshes can experience hydroperiods similar to those of associated salt marshes, but the flooding waters are fresh rather than salt. Upland runoff and precipitation also contribute to the hydrological budget of these wetlands, but their relative contributions can vary considerably from site to site. Vegetation species diversity tends to be much higher than in salt marshes. The natural vegetation is usually dominated by several broad-leaved plants such as spatterdock (*Nuphar luteum*) and pickerelweed (*Pontederia cordata*) and by wild rice (*Zizania aquatica*) and giant cutgrass (*Zizaniopsis miliacea*) in the lower portions. Cattails (*Typha* spp.), smartweeds (*Polygonum* (*Persicaria*) spp.), rosemallow (*Hibiscus moscheutos*), and others [45] can dominate the upper portions.

Common invertebrates in freshwater tidal wetlands include caridean shrimp (*Palamonetes* spp.), river shrimp (*Macrobrachium* spp.), the introduced Asiatic clam (*Corbicula fluminiea*) in the southeast, and some of the more motile estuarine invertebrates such as blue crabs (*Callinectes sapidus*), mud crabs (*Rhithropanopeus harissii*), and fiddler crabs (*Uca* spp.). In tidal freshwater wetlands, freshwater, estuarine, and anadromous fish species can be found. Among the freshwater group, cyprinids, centarchid, and ictalurid fish predominate [45]; representative species from the three groups are respectively, the spottail shiner (*Notropis hudsonius*) and silvery minnow (*Hybognathus regius*), bluegill (*Lepomis macrochirus*) and pumpkinseed (*Lepomis gibbosus*), and channel catfish (*Ictalurus punctatus*) and brown bullhead (*Ameiurus nebulosus*). Common species among the estuarine group are the mummichog (*Fundulus heteroclitus*), bay anchovy (*Anchoa mitchilli*), tidewater silverside (*Menidia peninsulae*) and hogchokers (*Trinectes maculatus*). Important anadromous species include blueback herring (*Alosa aestivalis*), gizzard shad (*Dorosoma cepedianum*), and sturgeon (*Acipenser* spp.). Sturgeon populations have been decimated by overfishing to the point where the shortnose sturgeon (*Acipenser brevirostrum*) is an endangered species, and the Atlantic sturgeon (*Acipenser oxyrinchus*) is very rare. Several marine fish species, including the Atlantic menhaden (*Brevoortia tyrannus*) and summer flounder (*Paralichthys dentatus*) may use tidal freshwater wetlands as nursery areas. In the south, these nursery areas are very important for snook (*Centropomus undecimalis*) and tarpon (*Megalops atlantica*) populations.

Because of their structural diversity, freshwater tidal wetlands are the home of a large number of bird species including wading birds waterfowl, shorebirds, raptors and passerines. Mammalian residents of these wetlands include meadow voles (*Microtus pennsylvanicus*), marsh rabbits (*Sylvilagus palustris*), beaver (*Castor* spp.), muskrats (*Ondatra zibethicus***)** and otter (*Lutrinae* spp.). Larger mammals such as deer (*Odocoileus* spp.) and bears (*Ursus* spp.) regularly venture into the wetlands in search for food and/or shelter.

As a result of historic development and land use patterns, this type of wetland has been reduced in coverage and subjected to degrading hydrologic modification. In more urban areas the wetlands are commonly degraded by the dominance of almost monotypic stands of non-native and/or invasive species such as *Phragmites* or *Typha* while in more rural areas they have been converted to agricultural production (e.g., rice, hay fields, *etc.*). Stormwater runoff from urban landscapes or agricultural fields further modifies the energy regime, freshwater supply, and nutrient/pollutant supply. Floodwater mosquitoes of the area will breed in these wetlands, often prolifically.

### 2.2. Freshwater (Non-Tidal) Wetlands

Freshwater (non-tidal) wetlands include a diversity of different plant forms, from forest-dominated through floating-leaved or submerged species primarily depending upon hydrology, topography, and soils. Under some situations, freshwater wetlands may include a mosaic of these wetland plant communities, while in other situations the wetlands may be more homogeneous in form, as in a forested swamp, pocosin, or emergent marsh.

The diversity of form and the widespread distribution of this wetland type make it difficult to standardize a policy or set of practices for mosquito management. A critical component of the mosquito producing potential for this type of wetland is the hydrological regime. Often, natural limits to mosquito populations prevail, including patterns of inundation and duration confined to cooler periods, the presence of natural predators in long duration water bodies, and others. In other cases, particularly in disturbed areas, mosquito production is significant enough to require control measures.

#### 2.2.1. Wet Meadows

Wet meadow marshes are seasonally wet with standing water, but typically drier than other wetlands, although the soil may remain saturated even when there is no standing water on the surface. They occur in poorly drained areas around lake basins and farmland, and high in mountainous areas. Hydrology is usually groundwater-driven, but the water table may lie from slightly above ground to more than 1m below the surface [47]. They are most common in the western U.S., Canada, and Alaska

Plant communities in wet meadows are highly seasonal depending upon water table depth. Vegetation in wet meadows includes sedges (*Carex* spp.), rushes (*Juncus* spp.), wildflowers such as marsh mint (*Mentha arvensis*) and smooth swamp aster (*Aster firmus*), and the marsh fern(*Thelypteris palustris*) [48]. Species with broad geographic range include various sedges (*Carex* spp.), tufted hairgrass (*Deschampia caespitosa*), Baltic rush (*Juncus balticus*), and Kentucky Bluegrass (*Poa pratensis*)*.* Trees (e.g., *Salix* spp.) may be abundant in circumscribed areas, but never widespread throughout the entire meadow.

Wet meadows support a number of amphibians and reptiles such as the northern leopard frog (*Lithobates pipiens*) and garter snakes (*Thamnophis sirtalis*), that feed upon abundant invertebrates. Many birds such as the northern harrier (*Circus cyaneus*), marsh wren (*Cistothorus palustris*), North American bittern (*Botarus lentiginosus*) live in or utilize this habitat for nesting or foraging. Common mammals include muskrat (*Ondatra zibethicus*), meadow vole (*Microtus pennsylvanicus*), and ermine (*Mustela ermine*); large mammals such as elk (*Cervus canadensis*), black bear (*Ursus americanus*), and gray wolves (*Canis lupus*) often frequent this habitat.

Major environmental impacts to this ecosystem include interruption or modification of surface and groundwater flows (reservoirs, water diversions, ditches, roads, water pumping). Additional habitat loss/degradation can be attributed to widespread logging and urbanization, hay production and direct grazing of cattle [47]. Although there are no mosquito species specifically associated with wet meadows, most floodwater mosquitoes that occur in the area will breed there.

#### 2.2.2. Wet Prairies

Wet prairies are grassland ecosystems that occur in the floodplains of streams and rivers, in depressions, and along lake margins. They occur throughout the central mid-western United States, parts of Canada and Florida [49]. These ecosystems are lowlands with moist to wet soil throughout the majority of the year due to poor drainage, often with standing stagnant water. A high water table is characteristic of wet prairie sites with soil textures and landforms varying with geographic location. Hydroperiod is intermediate between wet meadows and marshes [13], with standing water occurring for shorter duration and frequency than in marshes. Wet prairies may receive water from intermittent streams as well as from ground water and precipitation. Northern prairie grasses include blue-joint grass (*Calamagrostis canadensis*), prairie cordgrass (*Spartina pectinata*) and sedges (*Carex* spp.), and in South Florida include maidencane (*Panicum hemitomon*), spikerush (*Eleocharis* spp.), beakrush (*Rhynchospora* spp.), and water dropwort (*Oenanthe javanica* [50]).

During the wet season, wet prairie animal communities consist of aquatic and semiaquatic species similar to those of sloughs and include beaver (*Castor canadensis*), eastern tiger salamander (*Ambystoma tigrinum*), midland brown snake (*Storeria dekayi*), eastern massasauga (*Sistrurus catenatus*), American bittern (*Botaurus lentiginosus*), mallard (*Anas platyrhynchos*), northern harrier (*Circus cyaneus*) and sedge wren (*Cistothorus platensis*). As water levels fall in the dry season, some of the aquatic animals are forced into the deeper pond areas. Particularly in Florida, water levels rarely drop a foot below the land surface except in abnormally dry years [51]. Wet prairies have suffered destruction and severe alterations since 1900. Approximately 1,300 km^2^ (500 mi^2^) have been destroyed in southern Florida alone [52]. These alterations have occurred through drainage, water impoundment, conversion to agriculture, and exotic plant invasion [53]. Restoration efforts, such as in the Everglades (Florida), on the Kuhl Century Farm (Minnesota), and at the Woolsey Wet Prairie Sanctuary (WWPS) (Arkansas) are being undertaken throughout the U.S. As with wet meadows, floodwater mosquitoes of the region will also breed in wet prairies.

#### 2.2.3. Potholes

The Prairie Pothole region of North and South Dakota, Wisconsin, Minnesota and the Canadian provinces of Manitoba, Saskatchewan and Alberta covers approximately 780,000 km^2^ [10] (Figure 1) and is considered one of the richest wetland regions in the world because of the abundance of shallow lakes, marshes and smaller wetlands located in rich soils and warm summer climates [54]. Pothole wetlands were formed by glacial action during the Pleistocene. The greatest abundance of potholes is found in moraines of undulating glacial till. Once the basins were sealed with finer silts, water retention created suitable depths for semi-aquatic plants. 

Surface water inputs to potholes is very limited by the dearth of connection to surface water sources, and groundwater inputs are limited by the low permeability of the region’s glacial tills (although some connection is usually present). The major water sources for these potholes are snow, summer rain, and snowmelt runoff [55]. Hydrological patterns of potholes are diverse, both in time and space. There are yearly fluctuations in water levels, as well as high between year variation depending upon precipitation. Individual wetlands vary in the timing and duration of surface flooding. Some may remain flooded only for a few weeks after snowmelt, while others may be seasonal (flooded until early summer) semi-permanent (flooded until late summer) and permanent (Flooded for most of the year)

One common characteristic of prairie pothole are the concentric vegetation zones thatreflect topography-associated water level fluctuations. The marsh edge is dominated by sedges (*Carex* spp.), grasses (*Spartina* spp.) and forbs and sometimes woody species (*Salix* spp. and *Populus* spp.). During drought, perennial species such as *Typha* spp., *Scirpus* spp., and *Sparganium eurycarpum* and annuals such as *Polygonum* spp., and *Cyperus* spp. propagate from the seed bank. During wet stages, annual species that require exposed substrate for germination disappear and submersed species submersed species such as *Potamogeton* spp. and *Najas flexilis* appear. The emergent perennials can persist during the wet stages, but after several years they disappear resulting in pond-like conditions with only floating and submersed plants remaining [18].

It is believed that 50 to 75 percent of all waterfowl produced in North America originates from the Prairie Pothole region. The area is also home to many priority bird species including Franklin’s gull (*Leucophaeus pipixcan*), yellow rail (*Coturnicops noveboracensis*), and piping plover (*Charadrius melodus*), Baird’s sparrow (*Ammodramus bairdii*), Sprague’s pipit (*Anthus spragueii*), Wilson’s phalarope (*Phalaropus tricolor*), marbled godwit (*Limosa fedoa*), and American avocet (*Recurvirostra americana*). These wetlands are also important in the migration routes of the Hudsonian godwit (*Limosa haemastica*), American golden-plover (*Pluvialis dominica*), white-rumped sandpiper (*Calidris fuscicollis*) , and buff-breasted sandpiper (*Tryngites subruficollis*) .

Representative amphibians include the barred tiger salamander (*Ambystoma mavortium*) and the Great Plains toad (*Anaxyrus cognatus*); reptiles the painted turtle (*Chrysemis picta*) and the smooth green snake (*Opheodrys vernalis*); and mammals the American mink (*Mustela vison*), the coyote (*Canis latrans*), and the prairie vole (*Microtus ochrogaster*).

Unfortunately, it is estimated that only about 10% of the original wetlands in the area remain [13]. More than half of the original wetlands were drained or altered for agriculture. Other impacts include pollution due to runoff and grazing. Major efforts to protect the remaining prairie potholes have progressed since the 1960’s by agencies such as the U.S. Fish and Wildlife Services, Ducks Unlimited and The Nature Conservancy [13]. In an effort to restore this wetland type, artificial potholes have been excavated or blasted, or existing potholes have been deepened or partitioned to enhance waterfowl habitat [56]. Mosquito species associated with prairie potholes include *Aedes campestris*, *A. dorsalis*, *A. flavescens*, *A. vexans*, *Anopheles earlei*, *Culex tarsalis*, *C. territans* and *Culiseta inornata* [57,58].

#### 2.2.4. Playas

Playas are ephemeral, depressional, recharge wetlands that occur primarily in the high plains (Texas, Oklahoma, New Mexico, Colorado, and Kansas) but are particularly abundant in the Llano Estacado (Staked Plains) region (Figure 1). There are approximately 65,000 playas along the Great Plains [59,60]. They range in area from less than 1 ha to more than 250 (average 6.3 ha) [18]. These shallow, seasonal wetlands capture surface runoff, can function in flood attenuation, provide water for irrigation of surrounding agricultural fields, and play a major role in the recharge of underlying aquifers [60]. They are often biodiversity repositories and serve as ecological refugia in the arid and intensively cultivated high plains.

Playas are not normally connected to stable water sources and can be appreciably salty due to accumulation of salts from the underlying sediments and subsequent evaporation. They receive most of their water from precipitation and surface runoff and lose water through aquifer recharge and evaporation [61]. They are usually dry during late winter, early spring, and late summer and may experience multiple wet-dry cycles during a single year.

Because of the highly fluctuating conditions, the flora is dominated by annuals and short-lived perennials and is highly dependent upon the seed bank, and upon germination and growth conditions [18]. Of the approximately 450 plant species reported from High Plain playas, only two, the spotted evening primrose (*Oenothera canescens*) and the big bract verbena (*Verbenea bracteata*), occur consistently in most areas [61]. At a particular site, one can expect approximately 13 different plant species to occur at a given time, and about 19 different species during the growing season [62]. With the exception of several species that occur only at the edges, (*O. canescens*, turkey tangle fogfruit (*Phyla nodiflora*), and alkali mallow (*Malvella leprosa*), plant zonation is not evident in Playas.

Playa wetlands can be extremely rich in wildlife as they are water sources in an otherwise dry landscape. They support numerous species of invertebrates that are especially important to migrating waterfowl and shorebirds during their long treks between wintering and breeding grounds. Playas also support significant complements of reptiles, and amphibians such as the plains spadefoot toad (*Spea bomifrons*); waterfowl such as mallards (Anas platyrhynchos) and pintails (*Anas acuta*); and many other bird species including bald eagles (*Haliaeetus leucocephalus*) and whooping cranes (*Grus Americana*). More than 50 mammal species utilize the playas [18]; Eastern cottontails (*Sylvilagus flroidanus*) and various rodents are most common. Larger mammals include feral hogs (*Sus scrofa*) and mule deer (*Odocoileus hemionus*) Major threats to playa wetlands include sedimentation and contamination from surrounding agricultural runoff, urbanization, overgrazing, deliberate filling, and water diversion for agriculture. There is evidence of pesticide contamination of playas [63,64] including by some pesticides used for mosquito control [65], some at concentrations within the LC-50 range of various aquatic invertebrate species [66]. However, cropland agriculture, and associated pesticide use, predominates in the drainage areas surrounding playas and no direct linkages between pesticide levels and specific pest control applications have been established [64]. Some playa wetlands are protected due to their wildlife value and many neighboring farmers are adopting more ecologically sound farming techniques such as installing natural vegetation buffers, in recognition of the importance of playa wetlands.

Although many species of mosquitoes can develop in playa wetlands, the most common ones are *Culex tarsalis*, *C. quinquefasciatus*, *Aedes nigromaculis*, *Psorophora signipennis*, and *A. vexans* [67]*.* Large numbers of *Aedes* and *Psorophora* can be produced following the heavy rains of June or July, followed by increasing numbers of *Culex* as the emergent annual vegetation increases in the playa [68]. 

#### 2.2.5. Vernal Pools

Vernal pools, also known as woodland pools, are seasonally flooded depressions that are dry for part of the year (usually summer and fall). Individual pools are generally isolated, but several may be connected to each other through shallow swales. In the United States they occur primarily in the Pacific Coast and in the North and Northeast.

They rarely have groundwater inputs; they fill with rainfall, snowmelt, or runoff. A vernal pool may go through several cycles of filling and drying in one year, or may not flood at all during dry years. Although the underlying soil types vary, in most cases there is a hardpan layer which causes the retention of water in the pools.

Vegetation in vernal pools is highly variable and most pools are vegetated with widely distributed species. Individual pools may lack vegetation or may be vegetated with trees, shrubs, marsh and wet meadow species, aquatic plants or combinations of these [69].

Vernal pools provide valuable habitat to a number of rare species such as the San Diego mesa mint (*Pogogyne abramsii*), the longhorn fairy shrimp (*Branchinecta longiantenna*), and Swainson’s hawk (*Buteo swainsoni*). Vernal pools also afford critical habitat in the life cycle of certain amphibians and invertebrates, and are used by many bird species as feeding sites. Because of the regular drying, they usually do not support breeding fish populations. Many species such as fairy shrimp (*Eubranchipus* spp.), wood frogs (*Rana sylvatica*), and mole salamanders (*Ambystoma* spp.) are considered vernal pool indicator species because they are obligate or semi-obligate users of this habitat. 

As with other wetlands, considerable vernal pool habitat has been lost through urbanization, draining, conversion to permanent fish ponds, filling for mosquito control, conversion to agriculture, and many other activities [70]. Because of their small size and dispersion, the amount of habitat lost and still existing is not really known. 

Although predatory fish are usually absent, other larval mosquito predators such as odonates occur in the pools. Nevertheless, mosquitoes such as *Aedes canadensis*, *A. excrucians*, and *A. strictus* can often develop along changing borders, among vegetation, and in leaf litter and other detritus.

#### 2.2.6. Bottomland Swamps

Bottomland swamps are temporarily or seasonally flooded habitats that occur in the south-east and south-central United States. They are usually found along rivers and streams, but can occur in a variety of situations including valley bottoms, low-lying depressions, and areas of moisture-holding soils. In addition to providing important habitat for a large number of animal species, bottomland swamps play a variety of roles in the watershed including water storage and flood control and water quality improvement by removing excess nutrients, filtering sediments, and processing organic wastes. Bottomland swamps are also extremely productive, in part due to inputs of flood-transported nutrients and organic matter [71]. In North America, they are most abundant along the Atlantic coastal plain from Delaware to Florida, along the Gulf coastal plain to Texas, and along the Mississippi to Southern Illinois [72] (Figure 1).

They are normally flooded during most of the year, but water levels can exhibit significant fluctuations throughout the year and between years. Major water inputs include river overflow and runoff, but contributions from precipitation and ground water sources can be significant.

Bottomland swamps are deciduous forest wetlands usually dominated by various species of gum (*Nyssa* spp.), bald cypress (*Taxodium distichum*), and oak (*Quercus* spp.). Other tree species often occurring in these swamps include black willow (*Salix nigra*), red maple (*Acer rubrum*), river birch (*Betula nigra*), and sycamore (*Platanus occidentalis*). Shrubs may include buttonbush (*Cephalanthus occidentalis*), wax myrtle (*Myrica cerifera*), eastern swamp privet (*Forestiera acuminate*), and Virginia sweetspire (*Itea virginica*). Characteristic and secondary species, however, vary considerably depending upon slope, stream volume, soil type, flooding regime, and successional status.

Bottomland swamps support diverse aquatic invertebrate and fish communities many of which take advantage of floodwaters to disperse into the floodplain and exploit abundant food resources there [73]. They provide habitat to a number of species of special conservation concern such as the Seminole Texan crescent butterfly (*Phyciodes texana*), the Southern dusky salamander (*Desmognathus auriculatus*), the common rainbow snake (*Farancia erytrogramma*), the yellow-crowned night heron (*Nyctanassa violacea*), and the Louisiana black bear (*Ursus americanus luteolus*). Beavers (*Castor canadensis*) are important inhabitants of the swamps and are highly apparent due to their visible dams and associated flooded areas.

It is estimated that bottomland hardwood swamps once covered over 12 million ha across the south eastern and south central parts of North America, but only about 40% still remains [74]. A major factor in the coverage loss has been conversion to agriculture and forestry activities but losses to construction, flood control activities, reservoir construction, surface mining, and urban development have also been significant. Loss of this habitat has been partially blamed for the extinction or near extinction of several species including the ivory-billed woodpecker (*Campephilus principalis*), the Carolina parakeet (*Conuropsis carolinensis*), and Bachman’s warbler (V*ermivora bachmanii*) 

Mosquito species occurring in these habitats tend to utilize birds, small mammals, reptiles, and amphibians for blood feeding, but severe human pests such as several *Aedes* and *Psorophora* species can be abundant [75]. Common mosquito species found in bottomland swamps include *Aedes infirmatus*, *A. atlanticus*, *Psorophora* spp., *Anopheles crucians*, *Anopheles quadrimaculatus*, *Culex nigripalpus*, *C. erraticus*, *Culiseta melanura*, *Coquilletidia perturbans*, and *Uranotaenia* spp. Many of these species are known vectors of several arboviruses including those that can cause eastern equine encephalitis, western equine encephalitis and West Nile virus [27,75].

#### 2.2.7. Atlantic White Cedar Wetland Forests

Cedar-dominated wetlands are most commonly called cedar swamps or cedar bogs, with a variety of other designations restricted to specific regions (e.g., “spungs” in the New Jersey Pine Barrens, “juniper lights” in the Virginia-North Carolina Great Dismal, “juniper bogs” throughout the south). The native range of Atlantic white cedar (*Chamaecyparis thyoides*) is limited to freshwater wetlands along the Atlantic and Gulf coasts of the United States ranging from Maine to Mississippi [76] (Figure 1).

Hydrologic conditions are quite variable, but flooding usually occurs in late winter and early spring [18]. Cedar swamps situated in basins receive most of their water from precipitation, but others may receive significant ground water inputs. Their shallow, dark, generally acid waters are low in nutrients and are buffered by complex organic acids (e.g., humates, fulvic acids) [76] Surficial deposits beneath cedar forests provide groundwater storage and discharge and recharge areas. Peats adsorb and absorb nutrients and pollutants, purifying and protecting ground and surface water with which they are in contact [77].

Distinctive biotic assemblages grow under conditions too extreme for the majority of temperate-dwelling organisms. The shallow, dark, generally acid waters are low in nutrients and are buffered by complex organic acids (e.g., humates, fulvic acids). Surficial deposits beneath cedar forests provide groundwater storage and discharge and recharge areas. Peats adsorb and absorb nutrients and pollutants, purifying and protecting ground and surface water with which they are in contact. In many regions cedar wetlands are refugia for species that are rare, endangered, or threatened locally or nationally. The swamps form southern pockets for northern species at the geographic limits of their ranges, and similar northern pockets for southern species, while many locally common aquatic plants and animals are absent from cedar swamps [78,79].

Atlantic white cedar (*Chamaecyparis thyoides*) normally dominate the landscape, but other canopy species such as red maple (*Acer rubrum*) black gum (*Nyssa sylvatica*), sweet bay (*Magnolia virginiana*), and various pines (*Pinus* spp.) often co-occur. Open canopy stands usually have a well developed shrub layer that includes bitter gallberry *(Ilex glabra*), fetterbush (*Leucothoe racemosa*), swamp honeysuckle (*Rhododendron viscosum*), poison ivy (*Toxicodendron radicans*), poison sumac (*T. vernk*), and highbush blueberry (*Vaccinium corymbosu*) and cranberries (*Vaccinium* spp.).

In many regions cedar wetlands are refugia for species that are rare, endangered, or threatened locally or nationally. The swamps form southern pockets for northern species at the geographic limits of their ranges, and similar northern pockets for southern species, while many locally common aquatic plants and animals are absent from cedar swamps [78,79] They support numerous amphibians and reptiles such as the slimy salamander (*Plethodon glutinosus*), the eastern painted turtle (*Chrysemis picta*), the Southern copperhead (*Agkistrodon contortris*), and the timber rattlesnake (*Crotalus horridus*). These wetlands provide excellent habitat for deer, rabbits, and birds. Parulid warblers such as yellowthroats (*Geothlypis trichas*) and prairie warblers (*Setophaga discolor*) are common inhabitant of these areas and many other bird species including the barred owl (*Strix varia*), The sharp-shinned hawk (*Accipiter striatus*), and the purple finch (*Haemorhous* (*Carpodacus*) *purpureus*) frequent this habitat. White cedar foliage is a preferred winter browse for white-tailed deer (*Odocoileus virginianus*), and cottontail rabbit (*Sylwilagus floridanus*) and meadow mouse (*Microtus pennsylvanicus*) feed on cedar seedling, and in some areas, bear feed on berries from the shrubby understory [76].

There are 13 species in 4 genera of mosquitoes that utilize AWC wetlands as larval habitat [80]: *Aedes abserratus*, *A. aurifer*, *A. canadensis*, *A. excrusians*, *A. triseriatus*, *A. cinerius*, *A. vexans*, *Culex pipiens*, *C. restuans*, *C. territans*, *Culiseta melanura*, *Cs. morsitans* and *Uranotaenia sapphirina*. Among these species *A. canadensis* and *A. excrucians* are aggressive mammal biting mosquitoes that can create nuisance problems [81]. *Culex pipiens* and *C. restuans* are known West Nile virus (WN) vectors. This habitat also supports development of *Culiseta melanura*, the enzootic vector of eastern equine encephalitis (EEE) virus. *Culiseta melanura* larvae are found and often over-winter in crypts under the arching roots of mature cedars. Although *A. vexans*, *Cs. morsitans*, *A. canadensis* and *A. triseriatus* are believed to be capable of harboring EEE virus, only *Cs. melanura* is known to transmit EEE to humans. Passerine birds are important enzootic hosts for the EEE virus [82].

#### 2.2.8. Bogs and Pocosins (Including Carolina Bays)

A bog (synonymously referred to as mire, moor and muskeg) is a peat-accumulating wetland with no significant inflows or outflows. They are characterized by spongy peat deposits, acidic waters, and a floor dominated by a thick carpet of mosses, mainly sphagnum species [18]. Bogs receive all or most of their water from precipitation (termed ombrotrophic or “cloud-fed”) rather than from runoff, groundwater or streams. As a result, bogs are low in the nutrients needed for plant growth, a condition that is enhanced by acid forming peat mosses. Depending on their location in the landscape, which determines their development, bogs can be described as “valley”, “raised”, “blanket” and “quaking” bogs. Bogs generally form in one of two ways: as sphagnum moss grows over a lake or pond and slowly fills it (terrestrialization), or as sphagnum moss blankets dry land and prevents water from leaving the surface (paludification). Over time, many feet of acidic peat deposits build up in bogs of either origin. Water flowing out of bogs has a characteristic brown color from dissolved peat tannins. Bogs are widely distributed in cold, temperate climes, generally associated with low temperatures and short growing seasons where ample precipitation and high humidity cause excessive moisture to accumulate, mostly in the boreal regions of the northern hemisphere. In the U.S. bogs are mostly found in the glaciated northeast (New England, the Adirondack region of New York and Pocono region of Pennsylvania) and Great Lakes regions (Figure 1).

Bogs serve an important ecological function in preventing downstream flooding by absorbing precipitation and have been recognized for their role in regulating the global climate by storing large amounts of carbon in peat deposits. The unique and demanding physical and chemical characteristics of bogs result in the presence of plant and animal communities that demonstrate many special adaptations to low nutrient levels, waterlogged conditions, and acidic waters, such as insectivorous sundew (*Drosera* spp.) and pitcher plants (*Sarracenia* spp.). Bogs also support species like high bush blueberry (*Vaccinium corymbosum*), cranberries (*Vaccinium* spp.), Labrador tea (*Ledum groenlandicum*), cotton grass (*Eriophorum* spp.), and a number of protected plant and animal species. As the peat builds and the bog surface is further removed from the water interface or along the upland edges trees such as black spruce (*Picea mariana*) and tamarack (*Larix laricina*) may grow.

There is a number of pestiferous mosquito species associated with boreal wetlands and because of the short summer season, these mosquito species can emerge in very large numbers. These habitats may be commonly referred to as “bogs”, however, they tend to be more minerotrophic and productive than true bogs and may in fact be more likely to be classified as forest or shrub swamps. Perhaps because these peatlands are largely remote, undevelopable and sometimes vast, mosquito control efforts are, for the most part, nonexistent. Bogs, because of their acidic and nutrient-poor nature generally do not support mosquito larvae. However, one mosquito species, *Wyeomia smithii*, has adapted to develop in water collected in insectivorous pitcher plants. This species is not of public health significance.

Similar to bogs in their development, pocosins are densely vegetated evergreen shrub wetlands. These evergreen shrub and tree dominated landscapes are found on the Atlantic Coastal Plain from the Delmarva Peninsula to northern Florida, and are particularly dominant in North Carolina. The word pocosin comes from the Algonquin Native American word for “swamp on a hill”. Usually, there is no standing water present, but a shallow water table leaves the soil saturated for much of the year. Pocosins range in size from less than an acre to several thousand acres and in most instances are located between and isolated from old or existing stream systems. Because pocosins are found in broad, flat, upland areas far from large streams, they are ombrotrophic like northern bogs. Also like bogs, pocosins are found on waterlogged, nutrient poor, acid soils. The soil is often a mixture of peat and sand containing large amounts of charcoal from periodic burnings. Pocosins are subjected to fire about every 10 to 30 years because they periodically become very dry in the spring or summer. The fires are ecologically important because they increase the diversity of shrub types in pocosins. The most common plants in pocosins are evergreen trees (loblolly pine (*Pinus taeda*), red bay (*Persea borbonia*), and sweet bay (*Laurus nobilis*)), and evergreen shrubs (wax myrtle (*Myrica cerifera*), gallberry (*Illex glabra*), titi (*Cyrilla racemiflora*), fetterbush (*Leucothoe racemosa*), and zenobia (*Zenobia pulverulenta*).

Bogs and pocosins often harbor abundant invertebrate, particularly insect, communities. Bogs rarely harbor fish because of the acidic conditions, although small minnows (e.g., *Umbra limi*) can sometimes be found. Surprisingly, bogs and fens are home to abundant amphibian populations including the rare four-toed salamander (*Hemidactylium scutatum*) and the green frog (*Rana clamitans*). Few reptiles are common in bogs, but some, like the bog turtle (*Glyptemys muhlenbergii*), can often been found there. Over 100 bird species are known to breed in North American peatlands (bogs, pocosins and fens) and many more use the habitat during other parts of their life cycle [83]. Included are the white throated sparrow (*Zonotrichia albicollis*) and the palm warbler (*Dencroida palmarum*). Mammals are rarely abundant in peatlands, but small rodent populations often exist, and large mammals such as moose (*Alces alces*) and black bear (*Ursus americanus*) often frequent these areas, particulalry the edges (see also fens, below). Some pocosins are very large and difficult to develop, and so they remain largely undisturbed thereby providing sizeable tracks of undisturbed habitat for species like black bears (*Ursus americanus*) and the endangered red-cockaded woodpecker (*Picoides borealis*). About 1,400 square miles of undisturbed pocosins remain today. By comparison, more than 3,000 square miles were drained between 1962 and 1979. Historically, pocosins were mostly threatened by agriculture. Today, timber harvesting, peat mining, and phosphate mining join agriculture as the biggest threats to the remaining undisturbed pocosins.

#### 2.2.9. Fens

Fens are also peat-forming wetlands that are distributed mostly in the cool, boreal regions of the northern hemisphere. They are generally associated with low temperatures and short growing seasons, where ample precipitation and high humidity cause excessive moisture to accumulate. Fens are distinguished by their strong connection to ground water that receives nutrients from sources other than precipitation: usually from upslope sources through drainage from surrounding mineral soils and from groundwater movement. Depending on the underlying parent material fens may be acidic to strongly alkaline, the former being labeled a “poor” fen (more similar to a bog) and the latter often called “rich”, “marl” or “calcareous” fens. Fens differ from bogs because they have a ground-water discharge, are less acidic, and have higher nutrient levels. In North America fens are found in the glaciated mid-western and northeastern United States, the Great Lakes region, the Rocky Mountains, portions of the Appalachian Mountains and much of Canada [13,84].

Because of higher nutrients in the root zone than bogs, they are able to support a much more diverse plant and animal communities. Fens are often covered by grasses, sedges, rushes, and wildflowers. Depending upon acidity, the vegetation may be dominated by *Sphagnum* mosses in acidic areas, with sedges, shrubs, and dicot herbs often prevalent in neutral and alkaline areas. Examples of the later include tussock cottongrass (*Eriophorum vaginatum*), *Carex* spp, and heather (*Calluna vulgaris*) and Some fens are characterized by parallel ridges of vegetation separated by less productive hollows. The ridges of these patterned fens form perpendicular to the down slope direction of water movement. Over time, peat may build up and separate the fen from its groundwater supply. When this happens, the fen receives fewer nutrients and may become a bog.Because of habitat losses elsewhere, fens are becoming increasingly important habitat for moose (*Alces alces*), deer (Odocoileus virginianus), black bear (*Ursus americanus*), beaver (*Castor canadensis*), lynx (*Lynx* spp.), fishers (*Martes pennanti*), snowshoe hare (*Lepus americanus*), otter (*Lontra canadensis*), and mink (*Neovison vison*). Because of the less acidic conditions and connections to streams, fens support more fish species than bogs. Species such as pike (*Esox Lucius*), walleye (*Sander vitreus*), bluegill (*Lepomis macrochirus*), smallmouth bass (*Micropterus dolomieu*), brook trout (*Salvelinus fontinalis*), brown trout (*Salmo trutta*), and killifish may inhabit fens or connected streams. Fens also provide critical habitat to many species of birds including the greater sandhill crane (*Grus canadensis*), great gray owl (*Strix nebulosa*), short eared owl (*Asio flammeus*), sora rail (*Porzana Carolina*), and sharp-tailed sparrow (Ammodramus nelsoni). Fens provide important benefits in a watershed, including preventing or reducing the risk of floods, improving water quality, and providing habitat for unique plant and animal communities. However fens, like most peatlands, experienced a decline in acreage at a rate of about eight percent from 1950 to 1970, mostly from mining and draining for cropland, fuel, and fertilizer. Although mining and draining these ecosystems provide needed resources, a fen naturally requires up to 10,000 years to complete formation [85].

Since fens are hydrologically driven by a high, relatively stable ground-water table they seldom flood in a manner that would support mosquito production. There are references in the literature regarding “fens” and heavy mosquito infestations however the terms fen, bog and marsh are used interchangeably in the lay literature. The acidic conditions of poor fens could support species like pitcher plants (*Sarracenia purpurea*) which, although carnivorous, do provide larval habitat for *Wyeomyia smithii*. This species however, is autogenous (can develop eggs without a blood meal, see below) and not of medical or economic importance [86].

#### 2.2.10. Riverine, Riparian, Floodplain Wetlands

Riparian wetlands form adjacent to rivers and streams and some may occur near the coast. Soils tend to be alluvial, and are periodically or regularly flooded by upstream runoff, and underground flows. Riparian wetlands are important as filters of upland runoff before it enters rivers and streams.

Riparian wetlands comprise a wide variety of forms, from frequently wetted short duration forested floodplain through long duration emergent wetlands occurring in quiescent areas in large water bodies, and an incredible variety of conditions between these extremes. Many of these wetlands may be small and highly interspersed in the landscape, but many are extensive and cover thousands of contiguous acres.

Riverine wetlands are normally supported by river hydrology, and with the exception of man-made regulators of flow (e.g., dams, in-line and off-line storage reservoirs, *etc.*), are not problematic in the development of high mosquito populations levels. Soils tend to be alluvial, and are periodically or regularly flooded by upstream runoff, and underground flows. Riparian wetlands are important as filters of upland runoff before it enters rivers and streams (see also Bottomland Swamps). Riparian wetland areas are generally forested, narrow, and have only small areas of inundation, if present at all. These areas typically do not produce significant mosquito population densities. However, as a result of the diversity of these wetland types, their distribution through the built environment, and the presence of tree falls, water holding debris (e.g., tires), and other elements, these systems can produce pestilential mosquito populations. Rockpools within these areas flood intermittently and can produce mosquitoes such as *Aedes atropalpus* and *A. japonicus*. As a result of their diversity of form and hydrologic regime, widespread distribution, and small scale, it is very difficult to manage mosquito populations effectively.

Similar to both the riverine and riparian wetlands, the term “floodplain wetlands” represent a diverse range of wetland conditions, from bottomland hardwood forests with months of inundation to well drained herb and forb dominated systems that are infrequently inundated for relatively short durations. Floodplain systems can be major producers or several species of mosquitoes—in natural and modified settings. For example, river floodplains in Vermont often produce significant numbers of mosquitoes in the spring following snowmelt which can have major impacts on tourism as well as on livestock and public health. Similar situations arise along the Susquehanna and Delaware Rivers. Other good examples are the Sudbury and Concord River floodplains near the city of Boston, which are very productive *Aedes vexans* (a potential bridge vector for eastern equine encephalitis). These floodplain wetlands may be associated with uber rural landscapes where mosquito populations present no problem to ultra-urban systems where even relatively low mosquito population levels are seen as problematic. These factors often complicate planned approaches to mosquito management.

### 2.3. Wetland Complexes

Several regions in North America support very large wetlands complexes. Two of the most important ones are the Florida Everglades and the Mississippi Deltaic Plain wetlands. Because of their extent, complexity, and ecological and economic importance, we include below short descriptions of these areas. Other important wetlands complexes in North America include the Great Lakes wetlands complex, the San Francisco Bay marshes, the Hudson Bay Lowlands, and the Canadian Central wetlands (Figure 1).

#### 2.3.1. The Florida Everglades

The term Everglades does not identify a distinct habitat type, but defines a South Florida watershed, part of which is technically a wide and slow flowing river, that contains a mosaic of habitats some of which are wetlands. The major wetlands in the region include the Everglades proper, the Big Cypress swamp, and the coastal mangal of Florida Bay. In addition to wetlands, the region supports a variety of habitats including marine/estuarine, pinelands, and hardwood hammocks.

The Everglades is dominated by sawgrass (*Cladium jamaicensis*) with interspersed hammocks that support a variety of tropical and subtropical plant species including palms and hardwoods. This “River of Grass” once conducted the water flowing from the Kissimmee chain of lakes, down the Kissimmee River into Lake Okeechobee to its ultimate destination in Florida Bay. Originally spanning almost 28,500 km^2^, the Everglades now covers half that area due to habitat alteration for agriculture, development, and flood control (Figure 1). West of the sawgrass Everglades is the Big Cypress Swamp, which is dominated by cypress (*Taxodium*) species with interspersed pinelands and wet prairies. To the South, the mangrove forest of Florida Bay contain the three major mangrove species described above, red (*Rhizophora mangle*), black (*Avicennia germinans*), and white (*Laguncularia racemosa*), and common associates such as buttonwoods (*Conocarpus erectus* ).

 The Comprehensive Everglades Restoration Plan (http:///www.evergladesplan.org) is an ambitious State-Federal initiative that aims to restore and protect the water resources of South Florida, including the Everglades. In spite of the fact that both local and national politics have caused complications in the implementation of various aspects of the plans, progress is steadily being made. The very steep price tag of the project (over US$13 billion), however, also means that implementation will surge and wane depending upon the economic situation and mood of the country. There are 43 mosquito species reported in the Everglades National Park, with the salt marsh mosquito *A. taeniorhynchus* being one of the most troublesome for humans. Other species commonly found include *C. nigripalpus* and *Wyeomyia* spp. [87].

#### 2.3.2. The Mississippi Deltaic Plain Wetlands (“Louisiana Wetlands”)

The Mississippi Deltaic Plain region is the largest wetlands system in the United States, with an extension of nearly 7,250 km^2^. Wetlands in this deltaic plain complex are often referred to as the “Louisiana Wetlands” [88] (Figure 1).

The Mississippi Delta plain is composed of six major drainage basins, each of which represents shifts (in time) of the major distributary of the river [89]. The Atchafalaya basin is the youngest and receives approximately one third of the flow from the Mississippi and Red Rivers. The large input of fresh water and the shallow depths result in a predominance of freshwater marshes. The next youngest is the current Mississippi River Delta which receives approximately two-thirds of the river’s flow. The associated wetlands are also mostly fresh, but there are brackish marshes along the edges. The Barataria, Terrebonne, Vermillion-Cote Blanche, and the Ponchartrain-Lake Borgne basins, are increasingly older and support extensive salt, brackish, and freshwater marshes, as well as forested wetlands along the upland edges.

Vegetation in these marshes is fairly typical of the specific marsh types elsewhere. In the salt marshes, smooth cordgrass (*Spartina. alterniflora*) and saltmeadow cordgrass (*Spartina patens*) predominate, and black rush (*Juncus roemerianus*), saltgrass (*Distichlis spicata*), and turtleweed (*Batis maritima*) occur in varying degrees of abundance. The same species occur in the brackish marshes, with *S. patens* more dominant than in the saline marshes and Schoenoplectus americanus (chaormaker’s bullrush) becoming common in some areas. *Panicum hemitomon* (maidencane), *Sagittaria falcata* (duck potato, bulltongue) several spikerush (*Eleocharis*) species, and *Alternathera philoxeroides* (alligatorweed) dominate the freshwater marshes, and often there is a transitional zone between these and the brackish marshes where *Phragmites australis* (common reed), *S. falcata* (duck potato) and *Bacopa monnieri* (water hyssop) are most abundant [58]. In what is left of the forested wetlands, cottonwood (*Populus deltoides*), silver maple (*Acer saccharinum*), and box elder (*Acer negundo*) are the predominant canopy species.

Because of extensive development along the Mississippi River, these wetlands are very vulnerable to human impacts. In addition to pollution and extensive habitat loss to residential, commercial, industrial, and agricultural development, artificial channeling and other modifications for flood control and irrigation have decreased the amount of sediment reaching the wetlands. The reduced sedimentation, and both natural and accelerated subsidence caused by extraction of oil and gas, and withdrawal of groundwater, have resulted in wetlands losses of 65–100 km^2^ per year [90]. The U.S. Geological survey estimates a loss of over 500 km^2^ of coastal wetlands due to Hurricanes Rita and Katrina [91]. However, the hurricanes may have also had beneficial effects by stabilizing parts of the coastline via the deposition of tons of silt and sediments [92].

Mosquito communities in these wetlands also resemble those of southern fresh-salt water marshes, with *Aedes taeniorhynchus* and *A. sollicitans* predominating in the salt marshes and in the brackish areas along with several *Culex* species such as *C. salinarius* and *C. nigripalpus*. In the freshwater areas, several *Aedes* species including *A. infirmatus*, various *Psorophora* species, *Anopheles crucians*, *Culiseta melanura*, *Coquillettidia perturbans, C. nigripalpus,* and several others can become abundant.

### 2.4. Constructed Wetlands

Constructed wetlands are man-made aquatic systems that are designed to carry out wastewater treatment. Municipal wastewater is most commonly treated by these systems; however, constructed wetland systems have been used to treat a variety of wastewaters including urban stormwater, agricultural wastewater, mine drainage, aquaculture wastewater, and industrial wastewater [93]. Constructed wetlands are categorized broadly into two types: free water surface (FWS) wetlands and subsurface flow (SSF) wetlands [94]. Most FWS wetlands contain rooted emergent vegetation inundated with standing water and are similar in appearance to natural marshes. FWS systems that utilize only submerged or floating vegetation are comparatively less common [93]; however, large FWS wetlands usually support a diverse flora with vegetation of more than one growth type [95].

SSF wetlands typically consist of a bed of porous material which is underlain by an impermeable layer such as a synthetic membrane or natural material such as clay. While these systems often also include plants rooted in the treatment matrix, the water table is maintained below the surface of the bed. The pattern of water flow through SSF systems can be horizontal perpendicular to the inflow and outflow water control structures, vertical moving either up or down through the permeable treatment matrix, or a mixture of vertical and horizontal flow regimes [94,96]. Mosquito production from SSF systems is usually not a concern unless clogging of the permeable medium occurs or the inflow rate exceeds the water-holding capacity of the wetland system and water subsequently pools above the surface of the bed.

A third type of constructed wetlands, which is not usually included in the engineering literature that focuses on freshwater treatment systems for improving water quality, is tidal-flow wetlands. Tidal-flow wetlands represent a hybrid approach between a natural wetland and completely man-made wetland. Tidal-flow wetlands are impoundments built to surround brackish wetlands such as coastal salt marshes and mangrove swamps that are flooded for extended periods with a primary goal to reduce the production pestiferous floodwater mosquitoes [97]. Secondary-treated wastewater is used occasionally as a supplement to brackish water to inundate a subset of these wetlands. Retention of the wastewater provides a dual benefit of water quality improvement prior to draining seasonally. Rotational impoundment management (RIM) is used to allow tidal flow during a portion of the year and to dry the impoundment during other times annually [98].

Depending on the design, water and vegetation management, and water quality, mosquito production can differ appreciably among FWS and tidal-flow wetlands. Constructed wetlands receiving low quality wastewater can produce large numbers of mosquitoes. Because treatment wetlands, especially large wetlands, include a variety of habitats and vegetation types, the mosquito fauna can be quite diverse [95]. *Culex* species tend be dominant in constructed wetlands treating nutrient-rich wastewater *Cx. nigripalpus* [95], *Cx. erythrothorax* and *Cx. tarsalis* [99]. However, floodwater mosquitoes such as *Psorophora* spp. and *Aedes* spp. can be common in systems supporting habitat features that undergo frequent inundation and drying. Mosquitoes (*Mansonia* spp. and *Coquilletidia* spp.) with larvae that use their siphons to puncture macrophytes to obtain oxygen can also be prevalent at wetlands, especially when floating macrophytes or exposed root systems of emergent macrophytes are present. *Anopheles* spp. can be common in systems with comparatively low organic loading rates and especially when mats of filamentous algae proliferate [100].

Control of mosquito production from constructed treatment wetlands is accomplished by a combination of source reduction techniques, such as vegetation management, and utilizing design features such as deep water zones that reduce mosquito production (both discussed in more detail in section 5.3.2 and section 6.6, below), and application of mosquito control agents [101,102,103].

## 3. Mosquitoes

### 3.1. Mosquito Biology

**Figure 2:**
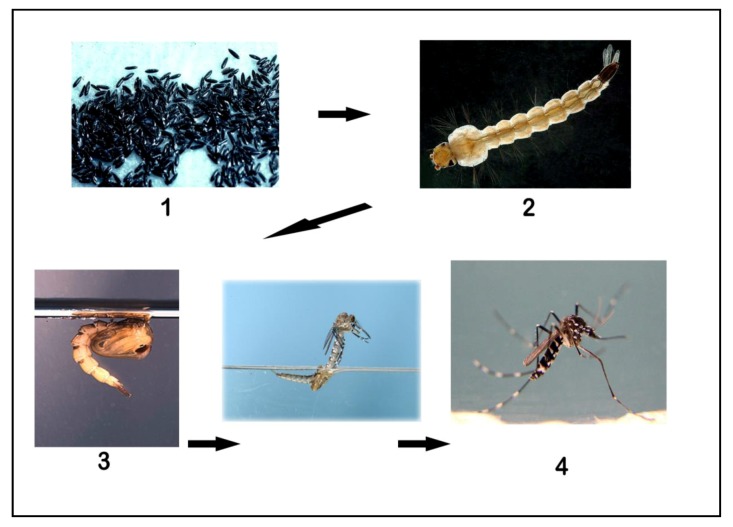
Mosquito (*Aedes* spp.) life cycle. 1. eggs, 2. larvae, 3. pupa, 4. adult. Adult emerging from the pupa is shown between 3 and 4.

Mosquitoes are true flies of the Order Diptera, with two wings and a pair of halters to aid in flight. These holometabolous insects develop through four morphologically distinct stages: egg, larva, pupa, and adult (Figure 2). Mosquitoes can be classified in two broad categories by the type of egg they lay. Floodwater mosquitoes lay eggs on moist surfaces, not standing water, and there is a drying out period that is required before the egg can hatch. Permanent water mosquitoes lay eggs on the water surface and will lose their viability if they dry out. Both floodwater and permanent water mosquitoes occur in wetlands.

The larval and pupal stages are strictly aquatic. The larval stage develops through four instars, each one progressively larger, feeding until the final days of the fourth instar. The pupal stage is different from many other insects in that they are active and swim when disturbed. The pupal stage is typically the shortest, often lasting just a few days.

Soon after emergence, mosquitoes begin flight activity. The males and females fly to seek sources of sugars for energy, consuming flower and plant nectars, honeydew, juices from decomposing fruits, and other sources [104]. After mating, the female mosquitoes feed on blood which is utilized to nourish the developing eggs. Some mosquito species can lay the initial egg batch without a bloodmeal, a biological function known as autogeny [105]. The first egg batch from a non-bloodfed female typically includes a small number of eggs relative to the potential. Once a bloodmeal has been consumed, egg batches can include as many as several hundred eggs.

### 3.2. Why Do Mosquitoes Bite?

The morphological structure of the female mosquito mouthparts allows them to pierce the skin of a blood host, probe for a blood vessel, and then imbibe blood. Prior to and simultaneous with the action of taking in blood, saliva is released from the salivary glands. If the salivary glands are infected with pathogens, the saliva can deliver those pathogens to the host.

Female mosquitoes feed on blood for the protein content. The protein is utilized to make yolk and to develop the eggs [106]. Some mosquito species are strict in their choice of a blood host. For example, *Culiseta melanura*, a species found in freshwater swamps, feeds primarily on avian hosts. Other species are generalists and will feed on various readily-available hosts. An example of an opportunistic species is the container mosquito *Aedes albopictus* which has been reported to feed on rabbits, deer, dog, humans, cats, squirrels, rodents, and avian hosts among others [107].

Generally, about three days is required for the fully engorged female to digest a blood meal. After this period, and after she lays the developing batch of eggs, she is ready to seek blood hosts once again. She will continue to consume blood meals and lay eggs for her entire life (7–60 days, normally about a month).

### 3.3. Mosquitoes and Disease

When female mosquitoes feed on blood, it is possible that they will feed on hosts that are infected with viruses and other pathogens. If the mosquito is a competent vector of a particular pathogen, after the infected blood meal is digested and an incubation period is completed, she can infect new hosts. She continues to bloodfeed for the remainder of her life and as the female mosquito ages, she becomes more dangerous, with the ability to infect new hosts simply by probing for a blood vessel and releasing saliva.

Pathogens of historical and immediate concern in North American wetlands include West Nile virus, St. Louis encephalitis virus, eastern equine encephalitis virus, western equine encephalitis, and the malaria *Plasmodium* parasite. Wetland vectors of importance in maintaining the cycle of these diseases include *Coquillettidia perturbans*, *Culiseta melanura*, *Anopheles quadrimaculatus*, *A. hermsi*, *A. freeborni*, *Aedes vexans*, *A. infirmatus*, *A. sollicitans*, *Culex quinquefasciatus*, *C. erraticus*, *C. nigripalpus* and *C. salinarius.* Some of these species feed primarily on birds and play a role in maintaining the virus in an enzootic cycle. Others are generalists that may be playing some role in the enzootic cycle, but serve a primary role in infecting mammals and other hosts outside of the wetland areas.

Some species are common to specific larval habitats, however, they are often found outside of what is considered their preferred water source. The table below (Table 2) provides a list of the North American mosquito species most commonly associated with the wetlands types described in this document.

**Table 2:** List of the North American mosquito species most commonly associated with the wetlands types described in this document. EEE = eastern equine encephalitis, SLE = St. Louis encephalitis, VEE = Venezuelan encephalitis, WEE = western equine encephalitis, WN = West Nile virus.

Mosquito Species	Habitat	Nuisance and/or Disease Associations	Associated Wetland Type	Range
*Aedes abserratus*	Freshwater cedar forests	Nuisance	Atlantic White Cedar wetland forest	North Atlantic Coast
*Aedes atlanticus*	Floodwater		Bottomland swamp	Southeastern US; Atlantic and Gulf Coasts
*Aedes atropalpus*	Floodwater		Riverine	Northeastern US, Atlantic Coast
*Aedes aurifer*	Freshwater cedar forests		Atlantic White Cedar wetland forest	North Atlantic Coast
*Aedes campestris*	Floodwater		Potholes	Northwestern US
*Aedes canadensis*	Floodwater: temporary shaded woodland pools and shaded pools adjacent to wooded areas	EEE, WN, dog heartworm	Vernal pools, Atlantic White Cedar wetland forests	Atlantic and Gulf Coasts, East of Rocky Mountains
*Aedes cantator*	Floodwater	Nuisance	Tidal salt marshes	Northern US
*Aedes cinereus*	Floodwater: freshwater cedar forests	WN, Nuisance	Atlantic White Cedar wetland forests, bogs and swamps	Atlantic Coast, Northern and Southeastern US
*Aedes dorsalis*	Floodwater: marshes and pools, overflow from wells	WEE, WN	Tidal salt marshes, coastal wetlands, potholes	Western US, Pacific Northwest, Northeastern US
*Aedes excrucians*	Floodwater: freshwater cedar forests	Nuisance	Vernal pools, Atlantic white cedar wetland forests	Atlantic and Gulf Coast, Northeastern and Northwestern US
*Aedes infirmatus*	Floodwater	EEE	Bottomland swamp, Mississippi deltaic plain	Gulf and South Atlantic Coasts, Southeastern US
*Aedes flavescens*	Floodwater	Nuisance	Potholes	Northeastern and Northwestern US
*Aedes japonicus*	Floodwater: containers, rock holes, shaded areas with water of high organic content	WN	Riverine	Eastern US
*Aedes nigromaculis*	Floodwater	Nuisance	Playas	Western US
*Aedes sollicitans*	Floodwater	EEE, nuisance	Mangrove, tidal salt marshes, tidal brackish	East and Gulf Coasts, Mississippi Deltaic Plain
*Aedes squamiger*	Floodwater	Nuisance	Tidal salt marshes, coastal wetlands	California, Pacific Northwest
*Aedes sticticus*	Floodwater	Nuisance, dog heartworm	Vernal pools	Eastern and Northwestern US
*Aedes taeniorhynchus*	Floodwater: salt marsh	Nuisance, dog heartworm	Mangrove, tidal salt marsh, tidal brackish, Florida Everglades, Mississippi deltaic plain	Atlantic, East and Gulf Coasts
*Aedes triseriatus*	Floodwater: treeholes, freshwater forests	Nuisance, EEE, WN, dog heartworm	Atlantic White Cedar wetland forest, Treeholes of deciduous trees	Eastern North America
*Aedes trivitattus*	Floodwater	Nuisance, dog heartworm	Meadows, swamps, woodlands	Eastern and Central US
*Aedes vexans*	Floodwater: freshwater cedar forests	Nuisance, EEE	Atlantic White Cedar wetland forests, Potholes, playas	Continental US
*Anopheles atropos*	Permanent water	Nuisance	Saltwater pools and marshes	Atlantic and Gulf Coasts
*Anopheles crucians*	Permanent water	Malaria	Bottomland swamp, Mississippi deltaic plain	Southeastern US; Gulf and Atlantic Coasts
*Anopheles earlei*	Semi-permanent and permanent water	Nuisance	Potholes, Bogs, marshes, woodland pools	Northern US
*Anopheles quadrimaculatus and sibling species*	Permanent Water: freshwater cypress swamps, river backwaters	Malaria	Bottomland swamp, Mississippi deltaic plain	Eastern US; Atlantic and Gulf Coasts
*Coquillettidia perturbans*	Permanent Water: Cattail ponds	EEE, nuisance	Tidal Freshwater (cattails), bottomland swamp, constructed wetlands, Mississippi, deltaic plain	Atlantic and Gulf Coasts, Eastern US; Northwestern US
*Culex erraticus*	Permanent Water: Freshwater cypress swamps	EEE, SLE, WN	Bottomland swamps	Eastern US; Atlantic and Gulf Coasts
*Culex erythrothorax*	Permanent water	WEE, WN	Constructed wetlands	West Coast
*Culex nigripalpus*	Permanent Water: ubiquitous in fresh water sources; sometimes found in brackish water	EEE, SLE, WN	Mangrove, bottomland swamp, Mississippi deltaic plain, constructed wetlands, Florida Everglades	Atlantic and Gulf Coasts
*Culex pipiens*	Permanent water, freshwater forests	WN	Atlantic White Cedar wetland forests, Constructed wetlands, playas	North Atlantic Coast, Northern US
*Culex quinquefasciatus*	Permanent water	SLE, WEE, WN, dog heartworm	Constructed wetlands, playas	Southern US
*Culex restuans*	Freshwater cedar forests	WN	Atlantic White Cedar wetland forests	North Atlantic coast
*Culex salinarius*	Permanent water	Nuisance, WN	Mangrove, tidal salt marshes, tidal brackish, Mississippi deltaic plain, constructed wetlands	Atlantic, Gulf and Pacific Coasts
*Culex tarsalis*	Permanent water	SLE, WEE, WN	Potholes, playas, constructed wetlands	Western and Southern US
*Culex territans*	Permanent water, freshwater forests	None for humans, feeds on cold-blooded vertebrates such as frogs	Atlantic White Cedar wetland forests, Potholes	North Atlantic Coast, Continental US
*Culiseta inornata*	Permanent water and temporarily flooded areas	WEE, WN	Tidal Salt Marshes (brackish upper marsh), Potholes	Atlantic and Gulf Coasts, Continental US
*Culiseta melanura*	Permanent Water: freshwater cypress swamps	EEE	Bottomland swamp; Atlantic White cedar wetland forests, Mississippi deltaic plain	Atlantic and Gulf Coasts; Eastern US
*Culiseta morsitans*	Freshwater cedar forests	EEE	Atlantic White Cedar wetland forests	North Atlantic Coast
*Deinocerites cancer*	Crab holes in tidal marshes	Nuisance	Tidal salt marshes	Eastern Coast of Florida
*Deinocerites mathesoni*	Crab holes	Nuisance	Tidal salt marshes	South Texas Coast
*Deinocerites pseudes*	Crab holes	Nuisance	Tidal salt marshes	South Texas Coast
*Mansonia dyari*	Permanent water	Nuisance	Constructed wetlands	Florida
*Mansonia titillans*	Permanent water	VEE, nuisance	Constructed wetlands	Florida to Texas
*Psorophora ciliata*	Floodwater	Nuisance	Rain-filled pools	Eastern US
*Psorophora ferox*	Floodwater	Nuisance	Woodlands, potholes	Eastern US
*Psorophora signipennis*	Floodwater	Nuisance	Playas	Central US
*Uranotaenia lowii*	Semi-permanent and permanent water; shallow margins of lakes	None for humans—Feeds on amphibians	Bottomland swamp	Southeastern US
*Uranotaenia sapphirina*	Freshwater Cedar forests, Permanent pools and ponds	None for humans	Atlantic White Cedar wetland forests, Bottomland swamp	North Atlantic Coast, Eastern US
*Wyeomyia smithii*	Pitcher plants	None for humans	Bogs, fens	Northeastern US
*Wyeomyia mitchellae*	Bromeliads	Nuisance	Florida Everglades	Southern Florida
*Wyeomyia vanduzeei*	Bromeliads	Nuisance	Florida Everglades	Southern Florida

## 4. Mosquito Control

### 4.1. Strategy

Mosquito control in wetlands can become necessary for a variety of reasons. Foremost among these is prevention of diseases caused by mosquito-transmitted pathogens such as West Nile virus, eastern equine encephalitis virus, and many others. More frequently, however, control is required because mosquito production from these areas can significantly impact quality of life and local economies [1] although there still exists a need to recognize that “quality of life” reasons for mosquito abatement are perfectly valid. Often local politics play a major role in mosquito control decisions.

Mosquito control, as a technology-based endeavor, should be science-based. However, in modern societies science is seldom applied in a vacuum, and social, political, economic, legal, and other considerations also contribute to decisions regarding the use of technologies, particularly in the public sector. These factors can influence mosquito control in different ways. For example, economic considerations may result in mosquito control being applied unnecessarily as when an important tourist or revenue-producing event is about to occur regardless of mosquito populations, or against a non-pathogen transmitting species when disease transmission has been reported in an area. Conversely, necessary mosquito control may sometimes be prevented by pressure from individual citizens or from local, regional, or national groups or organizations.

### 4.2. Surveillance

Responsible mosquito management should start with an effective surveillance program. We recognize two fundamentally different types of surveillance in mosquito management: (1) mosquito abundance surveillance, and (2) mosquito-transmitted disease surveillance. The former is usually carried out by professional mosquito control programs or local pest/animal control departments, whereas the latter is more often carried out by local, state, or federal health agencies. Here we will deal only with the first type. See Moore *et al.* [108] and Rutledge [109] for general information and examples of disease surveillance guidelines. Ideally, both types of surveillance programs should be integrated to formulate risk assessments that help make informed decisions for mosquito control before a public health emergency occurs.

The basic objective of a mosquito surveillance program is to establish baseline and contemporary data bases of mosquito population sizes and species composition, identify breeding/resting habitats, establish temporal and spatial patterns of abundance, and determine nuisance levels. Surveillance data are then used to evaluate the mosquito situation, to guide daily mosquito control operations including determining when and where treatment is necessary, and to evaluate the effectiveness of control measures. Inclusion of spatial data (mosquito-producing locations), physical data (e.g., temperature, rainfall, tide information, vegetation cover, *etc.*), and other variables known to affect mosquito ecology and behavior is often essential for an effective program [110]. Many mosquito surveillance programs also include an environmental monitoring component, particularly where habitat modifications, and/or water management for mosquito control are being employed (e.g., Boyce and Brown [111], Connelly and Carlson [97]).

Surveillance protocols should be tailored to local needs, and will differ depending upon local mosquito problems, habitats, climate, and resources. A clear definition of which local mosquitoes represent a potential problem (nuisance, economic, health, *etc.*) and monitoring the production and abundance of these species in space and time should form the foundation of mosquito surveillance programs. Successful programs incorporate immature and adult mosquito sampling to estimate mosquito abundance. Larval/pupal surveys are used to sample immature mosquitoes, whereas landing rate counts and a variety of trapping techniques are used to sample adult mosquitoes. Specific techniques for each will differ depending upon mosquito species, habitat, weather, *etc.* [112], but in general it is recommended that a combination of techniques be used to assure thorough sampling. Often, citizen reports/complaints can form an important part of the surveillance protocol and can help identify and temporally fill gaps in the program. 

Ideally, surveillance, environmental, physical, and spatial data should be integrated with control data in a geographical information system that allows near-real-time analysis of the various data sets and their interactions in a spatial context to help guide day to day mosquito abatement operations and evaluate their effects. In some cases GIS/GPS assisted surveillance and control operations can result in increased efficiency and precision, better mosquito control, and can provide automatic documentation for permitting and other reporting/regulatory purposes thus conserving resources and reducing the environmental impact of mosquito control [113].

### 4.3. Risk Assessment

Sound mosquito control strategies should originate from risk assessment procedures that clearly identify the (mosquito) problem, the possible mitigating (mosquito control) actions, and the anticipated adverse effects of each. 

The modern art and science of risk assessment originated in the 1970s from the urgent need to regulate pollution and has evolved considerably since then (see Callahan and Sexton [83] for a concise review). Two major types of risk assessment schemes that are directly relevant to mosquito control in wetlands are: *ecological risk assessment*—the process for evaluating how likely it is that the environment may be impacted as a result of exposure to one or more environmental stressors such as chemicals, land change, disease, invasive species, and climate change; and *health risk assessment*—the process used to estimate the nature and probability of adverse health effects in humans, domestic animals and livestock who may be exposed to chemicals (in this case mosquitocides) now or in the future. In the present context, health risk assessment also includes risk of infection with a mosquito-transmitted pathogen (see below). Mosquito control entities are also tasked with assessing the *nuisance/quality of life effects* of local mosquito production. 

Even though “risk” is not necessarily a quantifiable entity, most current risk assessment frameworks assume that risk can be measured and expressed quantitatively [114]. This is partly a result of legal considerations, particularly the 1980 Supreme Court’s “Benzene Decision” (Industrial Union Department, AFL-CIO *vs.* American Petroleum Institute) which emphasized the notion that quantitative demonstration of risk was a pre-requisite for regulatory intervention [115]. Particularly germane to this discussion is the fact that, often, the principal goal of mosquito control operations is to reduce mosquitoes to below nuisance levels, but we really do not have a clear definition of what those levels are [116]. Disease prevention is generally a much less frequent need in North America.

Formal risk assessment methodology is constantly changing in response to new technologies, evolving and emerging environmental challenges, and ever-changing societal pressures. Currently, issues such as challenges presented by climate change, new chemicals affecting endocrine system functions, new strategic societal priorities, and concern about cumulative risk assessment are forcing a re-examination of methods and techniques for formal risk assessment [114]. Many current risk analysis methodologies do not deal well with second, third, and higher order effects that may reinforce or offset the more obvious effects of a process or action under analysis [117]. 

Given the above, a formal qualitative risk assessment for mosquito control may not necessarily be desirable in many cases. Ideally, however, the following risk assessment and risk management issues must be evaluated before any *significant* wetlands mosquito control action is undertaken: 

Risk Assessment: (1) The mosquito production profile of the target wetland (based upon an effective surveillance program). (2) How local and regional wetlands fit into the overall mosquito problem. (3) What is the affected population (human and animal)? (4) What are the consequences of not managing mosquito production from the wetland in question? Risk Management: (1) When a mosquito problem becomes severe enough to warrant intervention that impact wetlands and/or human or animal health. (2) What potential control strategies are appropriate for the area? (3) What the anticipated impacts of the different control strategies are. (4) Can a step-wise or integrated approach be devised that employs only the minimum response necessary for a given situation? (5) How the performance of the control activities will be monitored and reported. (6) Design and implementation of an outreach campaign that engages locals and teaches residents (including public officials) about wetlands, mosquitoes, local control activities, and personal protection.

These and other issues, depending upon circumstances, should form the basis for a mosquito management plan for any given area that should be formulated *a priori* and that should consider routine as well as emergency management scenarios. This plan should be thought of as a work in progress, to be revised and modified as experience dictates or changing circumstances require. The plans should include guidelines and information on the surveillance and control of mosquito-borne pathogens, flexible risk assessment and decision support system models, a description of surveillance and control activities associated with virus transmission risk level, and elucidation of the roles and responsibilities of local and state agencies involved with mosquito borne virus surveillance and response [118]. Response plans for what are considered nuisance mosquitoes should also be developed. Ideally, such plans would allow vector control agencies and wetland managers to plan and modulate control activities to best represent the local conditions and surveillance methods as well as meet the objectives of wetland management. The efficacy of response plans to lessen mosquito related problems needs to be assessed.

More specifically, mosquito management plans incorporating adult mosquito control must be flexible to accommodate changing risk levels associated with decision-making criteria and to address unforeseen changes in factors not considered routinely as part of the decision-making criteria. The decision-making matrix of adult mosquito control programs often incorporates seven factors [111,119,120]: (1) initiation criteria, (2) treatment area delineation, (3) land use practices including the presence of sensitive species, (4) meteorological conditions, (5) continuance criteria, (6) termination criteria, and (7) additional factors that influence implementation of the plan. Additional factors could include unforeseen biological or environmental conditions in the wetlands, introduction of a novel invasive disease vector or pathogen, changes in legislation and/or regulations, availability of economic resources or equipment for mosquito control, availability of suitable adulticides, changes in the susceptibility of immature mosquito populations to larvicides (evolution of resistance), or a natural disaster. The thresholds for control actions are determined primarily by three factors: (1) initiation criteria trigger the initiation of adult mosquito control application measures; (2) continuance criteria trigger additional applications in an area that has previously attained an initiation criterion. These criteria are considered until a termination criterion is achieved for a treatment area. (3) Termination criteria include adult mosquito control application measures in a treatment area until initiation criteria are again met by the surveillance program. 

Thresholds for control actions will differ as a function of the surveillance methods being employed, the mosquito species, the landscape and location, presence of pathogens in the mosquito vectors, disease activity in the pathogen reservoirs and susceptible animals, as well as the aforementioned factors of the decision-making matrix. Thresholds for action incorporated into public health codes and regulations that must address mosquito production across all potential habitat types are stringent by necessity. For example, the presence of one mosquito larva in larval surveillance may be sufficient to trigger legal abatement proceedings. From a practical standpoint, the cost of abatement to achieve the elimination of all mosquitoes might be too high and the risk to public health or the nuisance activity from mosquito biting might be too low to merit intervention at this level. The immature mosquito abundance that triggers abatement efforts could range from an average of 0.2 for salt marsh mosquitoes, 0.25 mosquito larva/dipper sample for vectors of human malaria in floodplain wetlands and ricefields to 1.0 larva/dipper sample for vectors of arboviruses in wetlands of waterfowl hunting clubs. Treatment thresholds could be <0.2 larva/dipper sample for historically problematic mosquito developmental sites or where mosquito production per unit surface area is low but huge expanses of inundated habitat are present. These thresholds will be lower when pathogens are prevalent in the mosquitoes and/or disease activity is detected in sentinel animals and in wildlife, human and companion animal populations. Because the area of water in mosquito developmental sites often changes over time, the abundance of mosquito immatures in samples will increase as the water surface area decreases and concentrates the mosquito immature stages; a single threshold of immature mosquito abundance to trigger abatement activities is probably ill-advised under such conditions.

Likewise, the abundance of adult mosquitoes that triggers the initiation and continuance of abatement activities differs among habitats and ongoing pathogen activity. For example, in agricultural wetlands in the Central Valley of California, ≥100 female *Culex tarsalis* or ≥150 female mosquitoes of any genus (*Aedes*, *Anopheles*, *Coquillettidia*, *Culex*, *Culiseta*, *Ochlerotatus*, or *Orthopodomyia*) or ≥200 female mosquitoes in total collected during a single night or for three consecutive days by a carbon dioxide-baited trap will trigger abatement activities [80]. If New Jersey light traps (without carbon dioxide bait [81]) are used for adult mosquito surveillance, then only ≥10 female *C. tarsalis* or ≥25 female mosquitoes of any genus (*Aedes*, *Anopheles*, *Coquillettidia*, *Culex*, *Culiseta*, *Ochlerotatus*, or *Orthopodomyia*) or ≥50 female mosquitoes in total for three consecutive days (and nights) will trigger abatement activities. Moreover, if 1-minute sweep net samples or landing-count collections are used as the surveillance method, then ≥10 female *Aedes* or *Ochlerotatus* and/or ≥25 total female mosquitoes will trigger abatement activities [80]. If arbovirus activity is detected, then the treatment thresholds decrease by 75%. While 100 or more female *C. tarsalis* collected per trap-night in traps deployed at rice fields triggers abatement activities, a dozen individuals collected in a trap in an urban area could be indicative of the need for abatement activities in a wetland in a nearby park or in a riparian zone.

Establishing thresholds for mosquito control interventions is further complicated because, unlike agricultural systems where actual measurable indicators such as pest density or crop damage are directly relevant, individual perceptions and individual nuisance tolerance, both highly subjective and variable entities, must often be factored into mosquito control decisions. Furthermore, unlike agricultural crops, where economic losses due to pests are directly quantifiable in terms of crop losses and their corresponding dollar value, costs of mosquito infestations must be calculated in terms of quantities such as tourism and business losses, quality of life degradation, costs of human and animal diseases, and other equally indirect and often subjective variables.

Mosquito management when local arbovirus transmission is likely or has been demonstrated is a particularly tricky situation. First, the concept that only one or a few mosquito species are capable of transmitting a particular pathogen is not easily accepted by the public so during disease-transmission events mosquito agencies are often tasked with control of mosquito species that would normally not be targeted. Second, even though in many cases the individual probability of being infected, even during documented transmission events/outbreaks, is low [121,122,123], intensive vector control may still be required because even a single disease case is often deemed locally unacceptable, a resulting death unfathomable, and the social and economic ramifications of local disease transmission for the local community considerable [124,125,126,127].

### 4.4. Community Relations

An integral part of mosquito management is community outreach and education [128]. This activity includes three major components: (1) Public education about mosquito biology, ecology, and disease transmission; (2) Public outreach on local mosquito control operations; and (3) Continuous education of mosquito control workers on mosquitoes, mosquito control practices, and professional conduct. The first two help eliminate misconceptions about mosquitoes and mistrust of local agencies charged with mosquito management whereas the third makes mosquito control workers sources of information for the public and effective community ambassadors for their agencies. A better informed public will also be able to identify and eliminate sources of mosquitoes around the home, and take steps to reduce human contact with mosquitoes [129].

### 4.5. Personal Protection

In most pest control operations, a 90% reduction in the target pests is usually considered a success but in mosquito control, citizens can still be frequently bitten by mosquitoes after a 90% reduction in the mosquito population. Citizens must understand that personal protection such as the use of repellents and protective clothing, avoidance of mosquito producing habitats, reduction of outdoor activities during peak mosquito activity periods, and other precautions may still be necessary, particularly during disease transmission episodes.

## 5. Transient Methods of Wetlands Mosquito Control

### 5.1. Chemical Control

Use of chemicals for controlling wetlands mosquitoes should be undertaken only as part of an integrated pest management plan, and exclusive reliance upon pesticides is strongly discouraged. Chemical control of mosquitoes is by nature temporary and rarely 100% effective so it generally needs to be repeated in time to maintain adequate control. Insecticides can be applied to control mosquitoes during their immature stages (larvae and pupae—larvicides/pupacides) or during the adult stage (adulticides). It is generally agreed that larviciding is more effective than adulticiding because the aquatic immature forms are constrained within a water body where they are usually easily accessible, relatively immobile, and unable to escape. Flying adults on the other hand tend to be widely dispersed, often inaccessible, and highly mobile, and usually require adulticides to be applied over a larger area than larvicides. Furthermore, adults often tend to exist in closer proximity to humans than their larval counterparts [130]. Application of mosquitocides in wetlands should always be based upon conditions previously defined in a mosquito management plan, and documented via an effective surveillance program. Relative to other classes of pesticides such as those used in agriculture or horticulture, public health pesticides (including mosquitocides) are very limited in number. Pesticides for mosquito control occur in several chemical families, including organophosphates (e.g., Malathion), and pyrerthroids (e.g., permethrin). Other mosquitocides not easily classified by chemical family include insect growth regulators (IGR—e.g., methoprene), neonicotenoids and chloronicotinyls, microbial pesticides (e.g., Bti) and surface oils and films.

Larvicides can be applied using a wide variety of techniques including manually or with equipment mounted in trucks, boats, and all-terrain vehicles. The principal larvicides currently used in the U.S. for mosquito control include methoprene, several microbial agents, temephos, and various surface oils and monomolecular films. Methoprene is an IGR that mimics juvenile hormones of mosquitoes and other insects and prevents mosquito pupae from emerging into adults. It is effective in both fresh and salt water habitats. Microbial larvicides include *Bacillus thuringensis* var. *israelensis* (Bti), a naturally-occurring soil bacterium that is toxic to mosquitoes. The toxins are activated by gut enzymes of dipterans, particularly mosquitoes, black flies and midges so it is highly selective. Bti is effective in most habitat types. Another naturally-occurring bacterial larvicide is *Lysinibacillus* (formerly *Bacillus*) *sphaericus* (Ls) which is slower acting than Bti, but can recycle in nature and is thus more persistent.

Temephos is an organophosphate that is currently labeled for use in wetlands and has been used for mosquito control since the mid-1960s. Various types of surface oils have been used for mosquito control since the 1800s. They work primarily by suffocating the immature mosquitoes. Monomolecular films (MMF) are surfactants that reduce the surface tension of the water body and cause mosquitoes to drown. MMFs can also be effective against emerging adults and those that use the water surface for resting and drying their wings after emergence.

Mosquito adulticides are generally applied as low-volume sprays or ultra-low volume (ULV) mists. Product droplets sprayed by ULV equipment are very small and thus stay aloft for long periods, thus exposing actively flying adult mosquitoes to the pesticide longer than standard spray droplets. ULV equipment can also be mounted in a variety of terrestrial, aquatic and airborne vehicles. Thermal fogs, were commonly used in the past for both ground and aerial control of adult mosquitoes, but currently the method is only infrequently used to treat very small, usually indoor or peridomestic, areas via handheld equipment (e.g., [131]). Common products used for adult mosquito control include malathion and naled (organophosphates) and permethrin, resmethrin and sumithrin (synthetic pyrethroids). These chemicals are usually applied in very small concentrations thus minimizing non-target effects [132,133]. 

In addition to documenting the need for adult mosquito control and selecting the appropriate chemicals and equipment, application timing is critical for adult mosquito control because adulticides must be applied when mosquitoes are active and exposed. Generally, this is during dawn, dusk and early nighttime (19:00–24:00) hours. Different species of mosquitoes are active at different times (e.g., [134,135,136]) and environmental conditions also critically affect mosquito activity patterns [137,138,139,140]. Furthermore, it is essential to apply the chemicals under favorable weather conditions (*i.e.*, no rain, no thermals, wind speed <10 mph) to maximize efficacy and minimize drift of the product outside the target area.

As previously stated larvicides tend to be more focused in their application and are generally more specific than adulticides. Adulticides are applied over larger areas and have greater potential to impact other flying insects and non-target organisms. However, adulticides are applied at low concentrations of active ingredient and often at times when other organisms are not active thus minimizing exposure to non-target species. All larvicides and adulticides must be registered with the U.S. Environmental Protection Agency and state pesticide agencies before they can be applied. A series of rigorous studies must be conducted on the efficacy of the product, environmental fate, and impacts to other organisms before a product is considered for registration. There are also an extensive number of published studies by independent researchers on the laboratory and field efficacy of these products as well as impacts to non-target organisms. By and large, the vast majority of these studies conclude that, when applied at label rates and under favorable operational conditions, both larvicides and adulticides have negligible impacts on non-target organisms.

The National Pollutant Discharge Elimination System (NPDES) is a recent Federal permitting program that regulates point source discharges from pesticide applications to waters in the United States and requires all applicators to obtain a Pesticides General Permit (PGP) before applying pesticides. The system will greatly affect the application of chemicals for mosquito control to wetlands within the United States. There has been considerable debate over this issue as the regulations and requirements for application of mosquito pesticides to or over aquatic habitats are already covered by the Federal Insecticide, Fungicide, and Rodenticide Act (FIFRA).

The PGP requires all operators to minimize pesticide discharges (by using the lowest effective amount of pesticide, preventing leaks and spills, and calibrating equipment) and monitor for and report any adverse incidents. Operators who exceed the annual treatment area threshold must also submit a Notice of Intent (NOI) for coverage, implement integrated pest management practices to minimize the discharge of pesticides to waters of the U.S., develop a Pesticide Discharge Management Plan, submit annual reports, and maintain records of pest control practices.

Inquiries to mosquito control officials in Florida reveal that major impacts as of February 2012 include mostly time spent in compliance, which translates into manpower and additional expenses. Estimates of increased workload for implementation range from 5% to 20%. It is apparent from some of the comments that the NPDES will represent a greater burden to small mosquito control entities such as small municipalities or private organizations than to large ones. Also in some instances mosquito control entities are faced with compliance/coordination with several NPDES plans, for example, different county agencies may have their own plan which must be coordinated with the county’s plan, and perhaps with those of other county agencies. Several agencies reported that they had to invest in inexpensive GIS technology to automate the NPDES record-keeping requirements. None of the agencies contacted have experienced non-target impacts so the burden to mosquito control of that aspect of the new reporting requirements is not known. However, in some locations the cost of water tests and of monitoring for non-target effects has been substantial (pers. comm., David Brown—Manager, Sacramento-Yolo Mosquito and Vector Control District).

### 5.2. Biological Control

There are several biological agents that have been shown to cause mortality in mosquitoes including algae [141,142], oomycetes [143,144], bacteria (*Bti*, *Ls*, and recombinant bacteria) [145,146], microsporidian and gregarine parasites [147,148], pathogenic viruses [145], nematodes [149], predatory insects [150,151] including other mosquitoes [152,153], copepods [154,155], fish [156,157] and others. For general reviews of biological control of mosquitoes consult Chapman [158], Floore [159], and Rey [160].

One environmentally-attractive characteristic of biocontrol agents, particularly of pathogens and parasites, is their specificity. These organisms generally attack one or a few closely related species thus minimizing the potential for non-target effects. This specificity, however, also severely limits their potential market, which together with the usually high start-up costs for new products, often makes their commercialization difficult. Issues that need to be addressed when embarking on a biological control program against mosquitoes include the response of the biocontrol agents to pollution, their mechanisms for surviving drought periods, their method of recolonization of the target habitat following population crashes, their tolerance of pesticides including herbicides, and their anticipated interactions with local fauna.

Further difficulties with the use of biological agents include varying control efficiencies depending upon environmental conditions; the known dangers of introducing exotic species into nature or overusing native ones [161]; mass production, storage, and transport limitations; and lack of efficient delivery methods [158,159,162]. Other issues that must be considered when evaluating biological control of mosquitoes include determination that the anticipated reduction in mosquito populations will be adequate in terms of reducing nuisance/quality of life impacts and/or the risk of disease transmission [163,164,165] and if density dependent compensatory or overcompensatory responses to reduced larval density could result in no changes or even increases in the mosquito population as a consequence of attempted biological control [166].

For these reasons, biological agents (except for *Bti* and *Ls* formulations) will not be the exclusive method of mosquito control in the near future except in very small areas. Biological agents, however, should continue to form an integral part of IPM protocols for mosquito control (see below).

Without doubt, the mosquitofish *Gambusia affinis* and *G. holbrooki* are two of the most common biological control agents used for mosquito control but may pose problems if introduced as exotic fish (see later). These hardy species are extremely adaptable and efficient mosquito control agents if they have access to the larval habitats, and have been introduced for mosquito control throughout the world during the past 100 years [157,158]. Unfortunately, introduction of these species where they are not native can have devastating consequences to local ecological systems [167,168,169,170] so there continues to be an active search for local larvivorous fish species adapted to the appropriate habitats for use in mosquito control programs [157,171,172,173]. Mass production, storage, transport, and deployment of predaceous fish are areas where significant technological advances are still needed.

Cyclopoid copepods are another group of organisms recognized as mosquito predators [174,175]. They have proven extremely effective in eliminating *Aedes* mosquito production from container habitats such as water storage vessels and discarded containers [155,176,177]. Copepods are easy to mass produce and transport and can live for long periods in aquatic habitats [155]. They can survive on other prey when mosquito larvae are not available but the presence of alternate food does not significantly compromise their efficiency for mosquito control [178]. The efficacy of copepods in open water habitats such as wetlands is less well documented. They have been found effective in temporary pools and Louisiana marshes [154], rice fields [179], and in roadside ditches and some polluted habitats where larvivorous fish are lacking [180]. Use of fish is often more efficient in large open water habitats but copepods may still have a place to complement fish predation or in inaccessible areas or where fish are not present or hard to maintain [130]. As with other planktonic animals, the bodies of copepods can harbor disease-causing bacteria such as *Vibrio cholera* [181] and *Enterococcus* spp. [182] and some authors recommend filtering water containing copepods before drinking [183].

### 5.3. Habitat Management

#### 5.3.1. Water Management

High water flow rates and volumetric turnover rates can negatively influence mosquito production, but the flow rates needed to directly impact mosquito populations are too high to be of use as a sole technique for mosquito control (see below). Indirectly, however, water flow and turnover rates can impact mosquito populations by reducing or increasing stagnant pools of organic-rich waters that are attractive to certain mosquito species, particularly several *Culex* species [95,184] and by influencing water quality variables that are important for the survival of mosquito larval predators such as larvivorous fish and aquatic invertebrates. Various water manipulation techniques can also be used for vegetation management to eliminate some mosquito producing locations and to enhance predator access to others (see below). We note that wetlands hydrology is intimately connected with a multitude of ecological processes including vegetation composition; primary production; salinity and oxygen regimes; nutrient cycling; microbial dynamics; sediment transport; plankton, benthos, and nekton composition and population dynamics and many others [13,185,186,187]. Thus, artificial modification of wetlands hydrology should be undertaken with great care as it can severely impact wetland structure and function.

#### 5.3.2. Vegetation Control

Vegetation management, as it relates to mosquito control, is undertaken to create open water areas that are unfavorable for mosquito development or resting habitat [99] and to increase predation pressure on mosquito larvae [188]. This technique is most relevant to constructed wetlands, and is particularly important to consider during the design phase of the wetlands creation process. Vegetation provides food resources for mosquitoes in the form of plant detritus and also fosters the production of other mosquito food such as bacteria, algae, and protozoa [189,190]. Thick vegetation stands also may reduce water flow thus reducing physical disturbances such as high currents, eddies, and waves that can negatively impact developing mosquito larvae [190]. Reduction of vegetation coverage can significantly reduce mosquito populations [191,192,193], but the plant species, the method of vegetation thinning, and the spatial configuration of the remaining vegetation can have significant impacts upon the magnitude (or lack thereof) of the resulting mosquito population reduction [190]. For example, limiting vegetation coverage by using deep open water zones is probably more effective for mosquito control than periodic vegetation thinning [190,192,194]. Emergent vegetation usually recolonizes cleared areas quickly and there tends to be a flush of mosquito production after re-inundation of the marsh which offsets any reduction brought about by the ephemeral limitation of vegetative cover. Likewise, Lawler *et al.* [193] recommend using a combination of herbicides and disking rather than plain mowing (or grazing) due to rapid grow back of the vegetation. Although vegetation cover reduction can often be beneficial for wildlife and compatible with natural resource management goals [95,195], many important wetland functions such as water quality and wildlife habitat enhancement, are dependent upon wetland plant coverage, diversity, and productivity [9] and could suffer with reductions in vegetation cover. Once again flexibility, compromise, and effective mosquito surveillance are essential for environmentally sound mosquito control via vegetation management.

#### 5.3.3. Emergent Vegetation Control

A wide variety of techniques can be utilized for vegetation management in wetlands. These include selective removal (by plant species or marsh location), controlled burning [196], herbicide application, mowing and disking, grazing and other mechanical removal techniques [56,197], water level manipulations [198], and combinations of the above.

#### 5.3.4. Aquatic (Submerged and Floating) Vegetation Control

Several mosquito species, particularly *Mansonia* and *Coquillettidia* species use aquatic plants for oviposition and for respiration during immature development. Conventional mosquito control techniques such as surface films, larviciding, and biological control are usually not effective because of the great degree of shelter afforded by the usually thick mats of vegetation [199]. In such cases, vegetation removal or reductions in its coverage are the only alternatives to reduce mosquito populations. Mechanical harvesting of the vegetation using equipment such as aquatic harvesters, cranes, aquatic weed trimmers, and hand tools is routinely utilized, but coverage is limited to areas accessible to the equipment, and application to large areas can be expensive.

Herbicides can be used for aquatic plant management and can be applied aerially, from land-based, vehicle-mounted equipment, from boats, or “manually” using back-pack sprayers. The chemical herbicides are usually specific for one or a few aquatic plants. For example, Diquat is used against water lettuce (*Pistia stratiotes*), and 2,4 amine is used to control water hyacinths (*Eichhornia crassipes*, *Eichornia*. spp.).

Some biological control agents for aquatic plants such as the mottled water hyacinth weevil (*Neochetina eichhorniae*) and the water lettuce weevil (*Neohydronomus affinis*) have been utilized successfully in the past. The *Salvini*a weevil (*Cyrtobagous singularis*) has been effective in Australia [200], and several lepidopterans have been effective against milfoil (*Myriophyllum spicatum*; [201]), but large scale applications have been few and new agents and much more research are needed before the technique becomes reliable and cost-effective [202].

## 6. Long-Lasting Wetlands Modifications for Mosquito Control

Source reduction denotes techniques used to reduce mosquito populations by eliminating their oviposition and/or immature rearing sites, or by making habitats less conducive to mosquito production. Some of the techniques already discussed, such as aquatic vegetation control, are examples of source reduction techniques. In this section, however, we will deal with more enduring techniques. Long-lasting approaches to source reduction are often more economical than temporary control and if effective, eliminate or drastically reduce the need to use pesticides [203]. Some of these techniques, however, also have negative environmental impacts and tradeoffs will always need to be considered before their implementation in any given area. The most commonly used wetlands source reduction techniques include filling, ditching and runneling, impounding, open marsh water management, and the already mentioned topographic modifications to manage submerged and emergent vegetation, mostly in constructed wetlands.

### 6.1. Filling

In the past, filling wetlands to prevent mosquito breeding was an acceptable technique, particularly in wetlands close to populated areas or near new developments [11,204]. Since the “no net loss of wetlands” pledge by President Bush Sr., in 1986 it has become increasingly difficult to obtain permits for outright filling of wetlands for any reason and when they are issued they are accompanied by a mitigation requirement. The mitigation process, however, is highly flawed because of lack of oversight of the mitigation, in particular lack of criteria to evaluate mitigation success [205,206]. Because of the outright habitat and wetlands function losses, filling of wetlands for mosquito control is not considered an option in the present day.

### 6.2. Ditching

Ditching wetlands as a mosquito control technique has been in use at least since the turn of the 20th century [187]. Large wetlands expanses were ditched during the 1930s as part of work relief programs. This often included many low marsh areas that did not produce mosquitoes [207,208]. The standard procedure at the time called for shallow parallel hand-dug ditches (grid ditching) at approximately 200 linear m per ha [208]. Estimates of the extent of coastal wetlands ditching include over 90% from Maine to Virginia [209] and 94% along the New England coast [210].

In theory, ditching can reduce mosquito production by lowering water tables, reducing the number and size of water-holding depressions, and enhancing access by predatory fish to developing mosquito larvae. A properly designed ditch system can reduce the mosquito population using fish as biocontrol agents therefore reducing the need for pesticide intervention. Regular maintenance of the system will prevent the development of mosquito habitat, and the marsh can continue to be attractive to wildlife [211]. However, frequently, considerable mosquito production still occurred in grid ditched wetlands because ditches usually did not specifically target the mosquito producing areas [116]; considerable breeding still occurred in the areas between the ditches [11]; and many of the ditches quickly silted-in, sometimes creating isolated depressions where prodigious numbers of mosquitoes could be produced [207].

Ditching can profoundly change the nature of the impacted wetland [209]. By lowering the water table, ditching can transform a wetland into an upland habitat and can promote invasion by exotic plants [212]. Ditching can also significantly impact numerous wetland functions [187] and biota including vegetation [209], nekton, plankton, and benthos [187,213,214], terrestrial invertebrates [215], birds [216], and many others. Artificial ditching can also result in loss of open water habitat on the marsh surface [217]. Spoil associated with conventional ditching can be a significant impact on marsh ecology by interfering with natural marsh hydrology and providing additional substrate for upland plant species and weedy exotics [212]. In many areas, existing ditch systems must be maintained for a variety of reasons including water management, flood control, and mosquito control. For example, in Cape Cod, many (freshwater) ditches must be maintained by local mosquito control agencies where houses, roads, *etc.* have closed off creeks. These ditches are the only way water is carried from fresh to salt water. In some areas, mosquito control also assists with the maintenance of herring runs (pers. obs. GESH). Further, there are a large number of maintenance projects (with rakes or rotary ditcher) that are undertaken to maintain ditches on salt marshes. These projects limit the salt marsh mosquito populations leaving only small areas of larval habitat that can be treated with backpack sprayers using Bti. The local tidal tidal range is probably a huge factor in the success of the ditch maintenance program. Tidal ranges can be over 11 ft on a spring moon tide in coastal Massachusetts. Large tidal ranges drive the movement of water into and out of the marshes with significant momentum helping to keep outlets open and water levels low at the lowest tide cycles (pers. obs. GESH).

Ditching can also be used as a restoration technique for impacted wetlands. For example, in Florida, many coastal wetlands that were impounded for mosquito control have been re-connected to the estuary by using limited and directed ditches to control mosquito production [3,176,181]. These ditches are normally cut using rotary equipment that distributes the spoil as a very thin layer on the marsh surface. Although the remedy is not ideal, reconnection the adjoining estuary allows interchange of organisms and nutrients between both systems and often significantly improves environmental conditions in the former impoundment ([6], see below).

### 6.3. Runneling

Runneling is a technique developed in the 1980s and used predominately in Australia to control mosquitoes in intertidal salt marshes with no existing modifications for mosquito control. Runnels are very shallow (>0.3m) spoon-shaped channels that are constructed to follow natural water flow patterns [218]. The runnels are used to connect mosquito breeding depressions to a tidal source, thus increasing aquatic predator access to the developing larvae, and flushing out immature mosquitoes from the marsh before they can complete their development (see OMWM below).

Runneling has fewer environmental impacts than conventional ditching and can be very effective in controlling mosquitoes [219,220]. It is particularly effective in acid sulfate soils, where deeper substrate disturbance could create significant acidity problems (e.g., [221]). Because of the increased flushing, physical conditions in areas near runnels often resemble those of slightly lower areas [219]. Morton *et al.* [222] suggest that runneling actually enhances marsh-associated fish populations by increasing habitat access, and improving nutrient exchange between marsh and estuary. Chapman *et al.* [223] found little effect of runneling upon marsh crab populations except for a shift in species composition. Runneling can also sometimes lower water tables and salinities, particularly near the runnels [224]. Long term (>20 yrs.) effects tend to parallel those described above, and appear to be small, yet often statistically significant [225]. The maintenance costs of the runnels vary depending upon runnel system design and location [219], but are generally low [225].

### 6.4. Open Marsh Water Management (OMWM)

**Figure 3:**
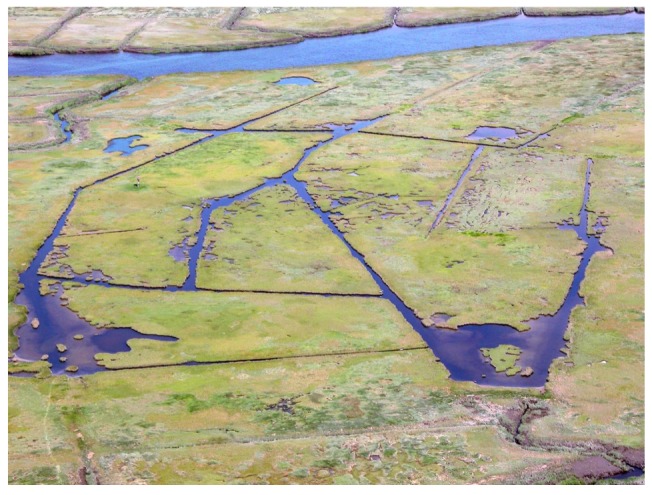
An OMWM project in Connecticut. Rotary ditches, ponds, and pond radials are visible.

OMWM is a technique developed in New Jersey in the 1960s for control of high salt marsh mosquitoes. It uses a variety of methods to eliminate actual mosquito breeding locations and to enhance tidal circulation, and/or improve predator access to others. Ponds or shallow pools (0.1–0.5 m) are constructed in areas with a high density of breeding depressions, and channels emanating from these (pond radials – similar to runnels) are used to connect other depressions in the vicinity of the ponds. Open (tidal), sill (semi-tidal), and closed pond and ditch networks ditches are used to eliminate mosquito breeding depressions and increase circulation throughout the system (Figure 3).

Spoil generated by the ditches and ponds is used to fill in other mosquito breeding depressions [10]. Originally, ditches and ponds were constructed by hand or via dragline [226] and the spoil had to be carefully spread and graded to avoid creating more mosquito breeding depressions or undesirable high areas in the marsh. At the end of a long hot day of ditching, the extra efforts associated with spreading out the spoil would be left until last and often forgotten. These retained spoil mounds paralleling the ditches reduced ditching effectiveness and supported the establishment of invasive plant species not initially present in the marsh. Later projects utilized rotary ditching equipment [227], which spread the spoil in a very thin layer over the marsh surface thus greatly reducing the need for grading and the creation of high points on the marsh surface, and facilitating natural re-vegetation of impacted areas [228]. Equipment malfunctions may sometimes also create low mounds paralleling the ditches, but when properly used, the technique can provide excellent mosquito control with a concomitant reduction or elimination of pesticides while minimizing negative impacts to marsh resources [229].

OMWM is frequently used by coastal mosquito control organizations along the Atlantic United States. The techniques used vary by locale due to hydrological/topographical differences at control sites [230]. For example, in some areas such as New Jersey and Delaware, standard OMWM modifications as described above are usually employed, but in some New England marshes, ditches are often plugged to create more long-lasting standing water areas [38]. Standards have been developed at the state level in New Jersey [231] and Delaware [107]. These standards have been modified by various mosquito control organizations along the coast and applied according to their specific requirements.

OMWM is very effective in reducing mosquito production from the affected area. Early studies documented significant reduction in mosquito abundance in OMWM modified marshes, and recent quantitative studies indicate significant reduction in mosquito production after application of OMWM techniques [38,232,233]. If properly applied, the technique can be compatible with other natural resource management/enhancement goals [233], and has been proposed by some as an element of more complex integrated marsh management techniques for mosquito control and natural resource enhancement [215].

There are also documented negative impacts of OMWM. For example, the technique may cause changes in soil surface moisture and water table [116,234,235] with accelerated rates of remineralization of organic detritus, effectively lowering the marsh surface [236] and negatively impacting some obligate marsh bird species due primarily to reduced forage effectiveness [237]. Other effects observed in wetlands subjected to OMWM include vegetation changes [238], loss of vegetation through subsidence and pond formation, increases in pH through oxidation of acid sulfate soils, degradation of water quality, changes in salinity [224,236], and shifts in the nekton communities from fish dominance to crustacean dominance [236].

### 6.5. Impounding

Wetlands impoundments have been used in the past for a variety of purposes including waterfowl management, water management and storage, flood control, agriculture, waste treatment, aquaculture, recreation, and many others [231]. The major North American salt marsh mosquitoes *Aedes* spp. will not oviposit upon standing water, so diking and flooding will effectively prevent mosquito breeding in the area. Only a thin film of water is necessary to prevent mosquito oviposition. A mosquito control impoundment is basically a marsh that has been diked (at least along the coastal edge) to allow the area to be flooded. A variety of water control structures such as pumps, culverts, and spillways can be used for water control. Management varies depending upon the location and other uses such as waterfowl management; some are flooded only during the mosquito breeding season and others may be flooded year-round or intermittently throughout the year [6].

An impoundment for mosquito control was constructed in Florida in the late 1930s [239,240] but was not very effective because of excessive water losses and no practical means of replacing them [241]. During the 1950s, impoundments (often constructed for other purposes such as salt-hay production) were managed effectively for mosquito control in the Mid-Atlantic States [242,243] and this encouraged the use of this technique in Florida, particularly along the Indian River Lagoon, in the east central part of the State. Ditches are not very effective in the coastal wetlands along this part of the State because lunar tides are very small or non-existent so there is very little natural energy to circulate water through ditch or OMWM systems. Also, the majority of the marsh acreage in this region is composed of high marsh, thus requiring modification of extensive areas for proper mosquito control.

**Figure 4:**
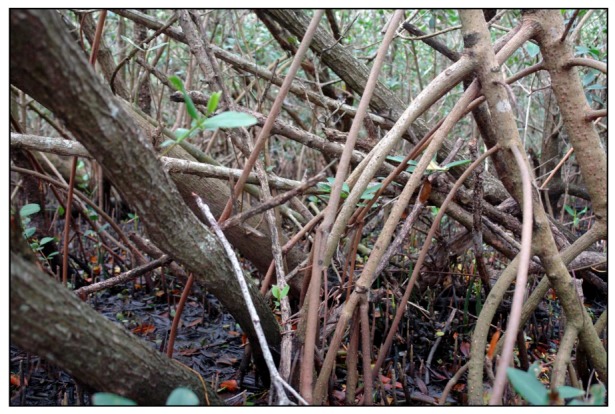
Florida (USA) mangroves illustrating their complex above-ground structure.

Finally, wetlands vegetation in the central and southern part of this region consist primarily of mangroves, whose complex above-ground structure (Figure 4) makes it difficult to construct ditches and ponds in the required locations. Impounding in this area commenced in 1954, and by 1970 over16,000 ha of coastal wetlands had been impounded for mosquito control [207,239].

Originally, impoundments were flooded much higher than needed for mosquito control to compensate for seepage and evaporation. The high flooding caused significant mortality of marsh vegetation including mangroves and herbaceous halophytes. Water quality in these early impoundments was also severely degraded [244,245] and plankton and nekton communities were severely impacted with drastic decreases in diversity and shifts in community composition [213,246,247,248,249,250,251]. Research on impacts of impounding in Florida and elsewhere led to reconnection of impounded wetlands, at least, during part of the year to the estuary through culverts, and the use of dedicated pumps to eliminate the need to overflood the areas. Many management modifications have been implemented to mitigate some of the adverse effects of impoundments [6,208]. Nevertheless, impounding requires a high degree of habitat modification and is not recommended unless needed for other management purposes such as waterfowl management.

### 6.6. Basin/Topography/Habitat Design

Constructed wetlands are effective means of water treatment and provide a number of ancillary benefits including provision of wildlife habitat, flood control, education and recreation opportunities, and aesthetic improvement of the landscape. These wetlands can also produce mosquitoes that will impact nearby human populations and resident wildlife. There are design elements that can be incorporated into a constructed wetland that will limit mosquito production and facilitate abatement efforts, but mosquito production is not often taken into consideration during the design phase of these wetlands [12].

Dividing a constructed wetland into cells (compartmentalization) to facilitate treatment by mosquito control equipment can significantly reduce mosquito abatement costs. Areas of deep water (>1m) limit the growth of aquatic vegetation, and reduce mosquito production by promoting waves and currents that limit oviposition and can drown immature mosquitoes and also enhance predation on mosquito larvae by fish and other aquatic predators. Although limited deep water zones are often recommended for constructed wetlands [101], deep water areas in general are less effective for water quality treatment than shallow vegetated areas.

Steep embankments also reduce mosquito production by reducing emergent vegetation, allowing better predator access to mosquito larvae, limiting the amount of habitat for floodwater mosquitoes created by water level fluctuations, and promoting wave action and currents that decrease habitat suitability for immature mosquitoes however, steep banks sometimes interfere with foraging by shorebirds. Bottom slopes must permit drainage without exposing the bottom to mosquito oviposition. Maintaining deep water zones and limiting emergent vegetation to raised plant beds can be effective in limiting mosquito production [192]. Growth of floating vegetation is sometimes a problem that can limit the effectiveness of these deep water areas for mosquito abatement [101].

As pointed out above, in many cases permanent elimination of emergent vegetation via topographic modification is more effective for mosquito control than repeated vegetation removal, and often reduces the disturbance-related invasion of exotics [9]. This technique however involves drastic modifications of wetlands and should be reserved for highly impacted areas, or for newly constructed treatment wetlands. In the case of the latter, if mosquito production concerns are addressed early during the design phase of a treatment wetland, the need for future interventions for mosquito control can be significantly reduced [102]. This is also true for wetlands restoration projects, but in this case the options for mitigating mosquito production by structural design means are severely limited.

## 7. Integrated Mosquito Management (IMM)

In most applications, the most efficient and ecologically sound pest control strategies consist of a combination of techniques that integrate public education, surveillance, chemicals (including repellents), biological control agents, cultural manipulations, habitat management, and others into a flexible scheme that produces the desired results with the minimum possible intervention [252,253]. Note that “minimum intervention” is not necessarily synonymous with less expensive as less intrusive techniques may often be more expensive to implement than more intense ones. Mosquito abatement agencies in the United States have been using integrated mosquito management practices for more than a century. Combinations of surveillance, sanitation measures, habitat management, chemicals, exclusion screens, education, and legislation were routinely used in many states in the early 20th century [254].

The major advantage of IMM is the reduction or elimination of pesticides with more benign alternatives. Other advantages include more effective mosquito control by use of complementary or supplementary techniques, ability to implement a tiered control approach depending upon need as determined by surveillance results, slower development of resistance to mosquitocides, reduced risk of chemical contamination of neighboring areas (including populated places), and many others. The major disadvantage of IMM is that it is a more complex process requiring more intimate knowledge of the habitat than simple chemical control. Other disadvantages include requirement of greater surveillance and record-keeping efforts, more intricate implementation, and sometimes higher overall costs.

## 8. Discussion

Mosquito control is an important part of the larger issue of wetlands protection and conservation. Agricultural, industrial, commercial, and residential development remain the major threats to wetlands, either as direct causes of outright habitat loss, or as primary causes of habitat degradation. Continued and increased legal protection of wetlands from the above forces remains the top priority, as without it, management related issues such as mosquito control will eventually become irrelevant and the much needed legislative mandates for responsible management of the country’s remaining wetlands unnecessary.

Although efforts at mitigating current wetlands losses appear to be effective, with more wetlands acreage created or restored than lost during the period 1996–2005 [255], the actual function of many of the rehabilitated/created wetlands is unknown, and the tangible gains in wetlands coverage are minute compared to historical wetlands losses. An example of an ambitious wetlands restoration program in North America is the reclamation of wetlands from agriculture to reduce nitrogen pollution and enhance carbon sequestration along the Mississippi-Ohio-Missouri basin [256,257,258]. Many restoration projects are directly associated with mosquito control; examples include hydrological reconnection of impounded wetlands along the Indian River Lagoon in Florida [6,208] and various salt marsh restoration projects in Connecticut and New York [253].

In spite of considerable progress during the last decades, habitat protection and environmentally sound habitat management still remain inextricably tied to politics and economics. Furthermore, the connections are often complex, and occur at several levels, ranging from local businesses and politicians, to multinational institutions and the Federal Government. Education is one of the keys to lasting wetlands conservation [259]. In the final analysis, politicians will do what will get them the most votes, and politicians, not wetlands scientists or public health professionals will decide the fate of this country’s wetlands. Unless we can produce a voting population interested in wetlands, the effective and lasting protection and restoration of these habitats will never be achieved. The current state of affairs, where the importance of environmental issues waxes and wanes depending upon a fickle electorate leads to a see-saw effect that is both inefficient and counterproductive.

The education of the American voters and future voters must be comprehensive so that regardless of the political climate and the Nation’s inclination, wetlands protection and restoration remains part of the National agenda. This means that the education process must influence a wide variety of individuals, of varying backgrounds, socioeconomic status, age, education, and national origins.

To achieve lasting results, the wetlands protection agenda must be based on science and fact. Untrue statements or misrepresentation of facts for political leverage or public relations effect are not helpful in the long term. This includes many commonly used assertions such as “healthy unimpacted wetlands do not produce mosquitoes” or “wetlands are disease reservoirs that without routine mosquito control would produce regular disease epidemics among human and animal populations”.

In reality, some healthy unimpacted wetlands can produce prodigious numbers of mosquitoes. One only has to read the accounts of the early Florida explorers that had to bury themselves in the sand at night to survive the onslaught of mosquitoes produced in Florida’s wetlands to appreciate this (e.g., [260,261]. And no, wetlands will not necessarily produce disease outbreaks if left unattended; many wetlands do not produce appreciable numbers of mosquitoes or disease vectors and in fact, it takes a rare combination of events involving, vectors, amplification hosts, pathogens, weather, and many other variables to produce an arboviral disease outbreak, and an even rarer combination to produce a real epidemic (although conditions may be more frequently met in other areas such as in malarious regions).

Unfortunately, such claims have become commonplace even among professionals because of the current politically-driven climate and because of the adversarial positions often existing between wetlands conservationists, developers, politicians, and mosquito control/public health workers. Such claims are counterproductive because they generate mistrust between mosquito control and wetlands professionals and politicians, public officials, and the general public. They also hinder cooperative work (and funding thereof) between wetlands ecologists and mosquito biologists that could produce more sound management of the Nation’s wetlands.

Two of the more difficult problems presently facing us are quantification of the nuisance mosquito problem, and private ownership of wetlands. The former is particularly vexing because different individuals have different tolerances for mosquito bites, and mosquito species vary in their aggressiveness when seeking blood meal, in their preferences for human blood, and in their flight ranges. Private ownership is a problem because it often interferes with establishment of best management practices in wetlands [6]. The purchase of privately owned wetlands by local, state, or Federal agencies is an obvious solution, but often funding is not available, property owners are not willing to sell, or they have unrealistic concepts of the true value of their land.

Because of the diversity of situations, there can be no hard and fast set of rules for mosquito control in wetlands. Several situations are clear, for example, failure to deal with vector mosquito production when significant arboviral disease transmission and amplification have been demonstrated near a populated area would be irresponsible, regardless of the overall probability of epidemic development. Likewise, undertaking permanent physical modifications for mosquito control of unimpacted or lightly impacted wetlands simply as a “precaution” is totally indefensible.

Most situations, however, are not as clear cut. Once political and popular support for responsible management of wetlands exists, many individual on-site determinations will still need to be made regarding wetlands management and mosquito control. This will necessitate working cooperation between mosquito control professionals, wetlands managers, and policy makers.

Some variables such as the type of wetland, land ownership, ecological status, location and size, surrounding habitats, proximity of human populations, mosquito production history, and others can be expected to be important in the management equation for most wetlands. Other variables however will be highly site and situation specific and will have to be factored in by professionals with local knowledge. A broad selection of management tools for the wetland professional is essential; this will allow development of flexible and site-appropriate management strategies for the Nation’s wetlands. 

In most cases, a surveillance-based tiered approach to mosquito control will be the best option for wetlands located near human populations. Whether within a framework of existing source reduction, personal protection, education, and community outreach programs, or where none exist, an escalating list of interventions may include no additional control when mosquito production is low, augmentation of biological control agents if mosquito nuisance levels are reached, limited use of biorational pesticides when nuisance levels become excessive, and comprehensive mosquitocide application when local disease transmission and amplification has been demonstrated.

Dogmatic investment in specific techniques for mosquito control is not conducive to a good final product and no viable strategy should be discounted a-priori on strictly philosophical grounds.

For example, in some cases a well-designed OMWM project may be the best solution for a particular wetland, however in other cases, OMWM may not be appropriate, modifications to existing OMWM systems may be desirable, or other means of mosquito control may be more suitable. Closures of certain wetlands to the public during heavy mosquito production times should be a viable alternative to heavy pesticide use or irreversible wetlands modifications when mosquito production from the site will not significantly impact densely populated areas. Economic impacts of such closures, (e.g., impacts to local livestock, tourist revenues, *etc.*) will need to be considered and will certainly be central to local political decisions.

One of the most pressing needs facing mosquito control workers and wetlands managers is the development of research-based guidelines for determining appropriate responses for different levels of nuisance mosquito production. This is a difficult task as a multitude of variables must be considered and some are difficult to measure/characterize. Included among these are the mosquito species, the type of wetland, the surrounding habitat and land use, the demographic characteristics and desires of the relevant human population, land ownership, weather conditions, local vector control capabilities, and many others. Development of defensible and transparent guidelines is further complicated by the fact that treatment criteria need to be based in part on the tolerances of the affected human population, and this can change with the make-up of the population and with local social and cultural characteristics that will influence a number of relevant variables such as the extent of exposure to mosquitoes of the population. A start would be development and field testing of protocols for quantifying impacts of nuisance mosquito population upon nearby human populations.

Development of operationally feasible and statistically valid methodology to quantitatively evaluate mosquito production is also highly desirable and applies directly to the above issue. Ideally, such a system would accurately reflect production from the study area (including spatial variation) and allow quantitative comparison of contemporaneous data with established standards for intervention (see above and section 4.3). Collins and Resh [262] discuss a well-grounded approach for nontidal wetlands based on sequential sampling techniques that had been previously used to sample mosquitoes in southeastern U.S. rice fields, and Walton [263] developed a sampling program using this approach to assess the effectiveness of BMPs for mosquito abatement in state-owned wetlands. Recent attempts to develop and apply statistically robust sampling methodology to wetlands mosquito populations include work by Rochlin and James-Pirri and collaborators [38,215,233]. Although the methodology used in such research studies is probably too cumbersome and expensive for routine mosquito control operations, they may provide a theoretical framework for developing more operationally friendly protocols.

A related urgent need is the development of reliable models of arboviral disease transmission that can provide decision makers with quantitative estimates of local disease risk given environmental conditions and measurable vector, host, and pathogen dynamics. Developing criteria and methodology for evaluating the accuracy and relevance of such risk models is an equally pressing need. Currently, most predictions of arbovirus transmission risk are based solely upon viral activity detection [264]. Some weather-based models exist that can call attention to conditions conducive to increased vector abundance, and weather based models have been used locally with some success, for example, to predict outbreaks of Ross River Virus (RRV) in Australia [265]. However, for most diseases current models based solely on weather and related variables have very limited potential for generating truly predictive output [264]. Independent models will likely be needed to accommodate the different ecological dynamics of different mosquito-borne pathogens [266].

Accurate information on the species and numbers of mosquitoes produced from different types of wetlands, deeper understanding of the factors that influence mosquito production in different habitats, and the spatial and temporal trends in mosquito production during wetland succession are also needed. Vezzani *et al*. [267] noted a dearth of information on immature mosquito habitats in the general scientific literature that is accessible to wetlands managers and policy makers. Some of this information may be available at local mosquito control agencies, but fine scale detail on the ecology of immature stages is still needed. Dale and Knight [224] point out that there are still large gaps in our knowledge of long-term effects of mosquito management activities upon non-target organisms and wetland functions. These authors also point out that our knowledge of the role of mosquitoes in the overall ecology of wetlands leaves a lot to be desired.

The number of products registered for vector control is small and dwindling. Many products are not being re-registered by the manufacturers because of cost of required tests relative to the amount of revenue generated by sales. Further research in needed to find new mosquito control products that are specific, effective and cost efficient, with negligible impacts to non-target organisms. Technological advances in the production, storage, transport, and deployment of biological control agents suitable for use in wetlands, as well as field studies on the efficacy, applicability, and environmental impacts of existing and novel biological control agents are also urgently needed.

Another crucial area in need of continued research is the anticipated impact of climate change upon wetlands ecosystems (and their management), upon mosquitoes, upon mosquito transmitted pathogens, and upon their interactions. Anticipated effects of climate change upon human health can be considerable and diverse [268], but our inability to estimate the nature, rate and extent of the anticipated change obstructs our ability to forecast the effects upon arthropod-borne virus systems and nuisance mosquito populations arising from wetlands. Direct impacts upon wetlands and mosquito ecology will obviously affect our overall wetlands management strategies, and our approach to mosquito management, but indirect effects upon vector-pathogen-host-environment systems, including socioeconomic and political repercussions, effects upon disease transmission, and many others complicate the issue by orders of magnitude. Research on controlling mechanisms of vector-pathogen-host interactions, and genotype based exploration of response patterns to environmental forcing are required to increase predictability of disease risk under different scenarios [269]. Broad environmental monitoring is generally advanced as a critical need for dealing with climate change [268], and this of course includes wetlands. Establishing baseline relationships between weather and disease is also considered essential in evaluating the possible effects of climate change upon all types of diseases [270].

## 9. Concluding Remarks

Presently, mosquito control is undertaken to protect public health and maintain expected quality of life. Mosquito control in wetlands is a complex issue influenced by numerous factors, including many hard to quantify elements such as human perceptions, cultural predispositions, and political climate. We have over 100 years of experience and of technological development that afford responsible agencies the capabilities to use integrated, surveillance-based approaches for controlling mosquitoes. These options, however, are context-dependent and the decision-making for abatement activities needs to be transparent and defensible.

Integrated mosquito abatement strategies incorporate many approaches and practicable options, as described herein, and need to be well-defined, effective, and ecologically and economically sound for the wetland type and for the mosquito species of concern. The approach will certainly differ in response to disease outbreaks caused by mosquito-vectored pathogens versus quality of life issues caused by nuisance-biting mosquitoes.

We encourage continued research on mosquito abatement techniques and on strategies and policies that enhance our ability to address wetlands mosquito production in an effective and ecologically sound manner.
